# The global economic burden of chronic obstructive pulmonary disease for 204 countries and territories in 2020–50: a health-augmented macroeconomic modelling study

**DOI:** 10.1016/S2214-109X(23)00217-6

**Published:** 2023-07-18

**Authors:** Simiao Chen, Michael Kuhn, Klaus Prettner, Fengyun Yu, Ting Yang, Till Bärnighausen, David E Bloom, Chen Wang

**Affiliations:** a Heidelberg Institute of Global Health, Faculty of Medicine and University Hospital, Heidelberg University, Heidelberg, Germany; b Chinese Academy of Medical Sciences and Peking Union Medical College, Beijing, China; c International Institute for Applied Systems Analysis, Laxenburg, Austria; d Wittgenstein Centre, Vienna, Austria; e Vienna University of Economics and Business, Department of Economics, Vienna, Austria; f Department of Pulmonary and Critical Care Medicine, Center of Respiratory Medicine, China–Japan Friendship Hospital, Beijing, China; g Department of Global Health and Population, Harvard T.H. Chan School of Public Health, Boston, MA, USA; h National Clinical Research Center for Respiratory Diseases, Beijing, China; i Chinese Academy of Engineering, Beijing, China

## Abstract

**Background:**

Chronic obstructive pulmonary disease (COPD) is the third leading cause of death worldwide and imposes a substantial economic burden. Gaining a thorough understanding of the economic implications of COPD is an important prerequisite for sound, evidence-based policy making. We aimed to estimate the macroeconomic burden of COPD for each country and establish its distribution across world regions.

**Methods:**

In this health-augmented macroeconomic modelling study we estimated the macroeconomic burden of COPD for 204 countries and territories over the period 2020–50. The model accounted for (1) the effect of COPD mortality and morbidity on labour supply, (2) age and sex specific differences in education and work experience among those affected by COPD, and (3) the impact of COPD treatment costs on physical capital accumulation. We obtained data from various public sources including the Global Burden of Disease Study 2019, the World Bank database, and the literature. The macroeconomic burden of COPD was assessed by comparing gross domestic product (GDP) between a scenario projecting disease prevalence based on current estimates and a counterfactual scenario with zero COPD prevalence from 2020 to 2050.

**Findings:**

Our findings suggest that COPD will cost the world economy INT$4·326 trillion (uncertainty interval 3·327–5·516; at constant 2017 prices) in 2020–50. This economic effect is equivalent to a yearly tax of 0·111% (0·085–0·141) on global GDP. China and the USA face the largest economic burdens from COPD, accounting for INT$1·363 trillion (uncertainty interval 1·034–1·801) and INT$1·037 trillion (0·868–1·175), respectively.

**Interpretation:**

The macroeconomic burden of COPD is large and unequally distributed across countries, world regions, and income levels. Our study stresses the urgent need to invest in global efforts to curb the health and economic burdens of COPD. Investments in effective interventions against COPD do not represent a burden but could instead provide substantial economic returns in the foreseeable future.

**Funding:**

Alexander von Humboldt Foundation, National Natural Science Foundation of China, CAMS Innovation Fund for Medical Science, Chinese Academy of Engineering project, Chinese Academy of Medical Sciences and Peking Union Medical College project, and Horizon Europe.

**Translations:**

For the Chinese and German translations of the abstract see Supplementary Materials section.

## Introduction

Chronic obstructive pulmonary disease (COPD) is the third leading cause of death worldwide, causing 3·3 million deaths in 2019.^1^ The global death toll of COPD increased by 14·1% between 2009 and 2019 because of factors such as urbanisation, air pollution, and tobacco use.^1^ The health burden of COPD is distributed unevenly between countries, with 90% of COPD-related deaths occurring in low-income and middle-income countries (LMICs), despite the fact that these countries only account for 83% of the global population.^2^ In 2019, China bore the largest COPD death toll in absolute terms, followed by India and the USA. South Korea had the highest COPD death rate, followed by Nepal, Denmark, and China.^1^ The incidence rate, prevalence, and mortality for COPD are shown in detail in appendix 3 (pp 2–3).

In addition to causing a massive health burden, COPD also inflicts a large economic toll. The lack of information about the economic burden of COPD contributes to the insufficient attention that governments and policy makers pay to this chronic condition.^3^ Ministries of health and health services, particularly in LMICs, and international organisations have proposed few health policies that are aimed at preventing COPD, and there is limited access to diagnostics and little effective therapy.^4^ Developing comprehensive projections of the disease's economic burden and how this burden is distributed across countries is an important prerequisite for the creation and implementation of evidence-based policies to decrease morbidity and mortality from COPD.

Although some studies have estimated the economic burden of COPD for one or a few countries, most studies are based on aggregating the estimated direct and indirect costs of COPD in different countries (the cost-of-illness approach), or on multiplying COPD cases and deaths by the willingness of individuals to pay to avoid risks (the value-per-statistical-life approach).^2^, ^5^, ^6^, ^7^, ^8^ However, these approaches do not account for economic adjustment mechanisms. First, they ignore that jobs do not remain vacant indefinitely in real-world economies because companies substitute lost labour with new workers or with physical capital (machines or robots). Second, these approaches are static and do not account for population dynamics as driven by morbidity-related and mortality-related changes, nor for the dynamics of physical capital accumulation as driven by COPD's implications for treatment costs and savings. Failing to account for these adjustment mechanisms generally leads to an overestimation of the economic burden of COPD from lost human capital. This is a common problem in projecting the economic burden of diseases and largely explains the high numbers usually found in cost-of-illness and value-per-statistical-life studies.^9^

**Research in context**

**Evidence before this study**

We searched for the terms “COPD” (or “chronic obstructive pulmonary disease”) and “economic burden” (including the variants “economic cost” and “economic loss”) in the titles and abstracts of papers included in MEDLINE, PubMed, and Google Scholar between June 1, 1960, and Dec 1, 2021, and in the references from relevant articles. The search was restricted to publications in English. Most previous studies aggregated the direct and indirect costs of chronic obstructive pulmonary disease (COPD; the cost-of-illness approach) or multiplied COPD mortality and morbidity by the willingness of individuals to pay to avoid risks (the value-per-statistical-life approach). However, both approaches ignore that jobs vacated due to illness or death do not remain vacant indefinitely because companies substitute lost labour with new workers or physical capital (machines or robots). In addition, both approaches are static and fail to account for the dynamics of morbidity-related and mortality-related population changes and for treatment costs and, thus, physical capital accumulation. Moreover, most previous studies only focused on a small number of countries (often only one).

**Added value of this study**

Using a theory-based simulation model to estimate the macroeconomic burden of COPD for 204 countries and territories, we found that COPD will cost the global economy INT$4·326 trillion (uncertainty interval 3·327–5·516; at constant 2017 prices) in 2020–50—nearly half of India's total gross domestic product (GDP) in 2019—which will be distributed unequally across regions and countries. The highest aggregate economic burdens will occur in China, the USA, and India, which have the three largest populations in the world. The per-capita burdens were highest in Ireland, Monaco, and the USA. In terms of share of GDP, the USA, North Korea, and Germany face the largest economic burdens. The economic burden of COPD was not distributed in proportion to population size or disability-adjusted life-years (DALYs): although low-income and middle-income countries accounted for an economic loss of INT$2437 billion (56·4% of the global total) from COPD, these countries represent nearly 90% of the global disease burden (in DALYs). Our findings fill several knowledge gaps. First, this study estimates for the first time (to our knowledge) the macroeconomic burden of COPD for all countries worldwide. Second, that high-income countries incur the highest macroeconomic burden of COPD in absolute terms, while low-income and middle-income countries bear the greatest health burden. Middle-income countries face the highest economic burden of COPD as a proportion of GDP. China alone accounts for 83·5% of the economic losses in upper-middle-income countries, despite having only 56·3% of their population.

**Implications of all the available evidence**

Our findings underscore the need to make improvements on multiple fronts, including strengthening health systems, implementing laws and public health policies to reduce tobacco use and air pollution, improving enforcement of existing laws, and raising public awareness. Without these improvements, an economic reckoning will accompany the existing human costs: the global economic burden of COPD could grow enormously in the coming decades. Urgent investment is needed in global efforts to curb COPD and its associated disease and economic burdens.

To our knowledge, no previous study has produced a comprehensive global estimate of the economic burden of COPD based on simulating the effect of COPD on the productive capacities of economies at the aggregate level. To fill this gap, we aimed to use a theory-based, health-augmented macroeconomic model to estimate the macroeconomic burden of COPD for 204 countries and territories from 2020 to 2050 (31 years in total) and to find its distribution across world regions. This approach has previously been used to assess the economic burdens of several non-communicable diseases, COVID-19, road traffic injuries, and risk factors such as tobacco and air pollution.^10^, ^11^

## Methods

### Model description

In this health-augmented macroeconomic modelling study, we estimated the macroeconomic burden of COPD for 204 countries and territories. COPD was defined according to the Global Burden of Disease (GBD) Study's COPD category.^1^ Of the 204 countries and territories, 144 had all the data inputs required for our projections. We directly calculated the macroeconomic burden of COPD for these 144 countries using the health-augmented macroeconomic model described in detail in previous studies^9^, ^10^ and in appendix 3 pp 3–7. The model accounts for the following channels of economic burden. First, deaths attributable to COPD reduce the number of individuals of working age, and COPD morbidity reduces productivity and increases absenteeism among workers. Second, households with an individual with COPD must divert resources from savings to finance out-of-pocket treatment costs. Alternatively, insurers pay for treatments and therefore must increase premiums, which implies the same effect. Irrespective of whether treatment costs are financed individually or via health insurance, treatment-related expenses reduce aggregate savings and investment and therefore hamper economy-wide physical capital accumulation. Note that shifts from consumption of other goods and services to health care do not constitute an economic loss but merely a sectoral reallocation of resources within an economy. This is because the shift from consumption expenditures to health expenditures does not reduce the productive capacity of the economy. By contrast, a shift of funds from investment in physical capital to the consumption of health care reduces future growth prospects.

To calculate the economic burden of COPD, we compared annual aggregate output in the form of gross domestic product (GDP) across two scenarios over the period 2020–50: (1) a status quo scenario, in which GDP is projected to grow on the basis of current estimates and projections of disease prevalence, and (2) a counterfactual scenario, in which COPD prevalence is eliminated from the beginning of the timeframe.

### Data sources

We used data from 204 countries and territories and a set of aggregated World Bank regions. The GDPs and saving rates (accessed April 25, 2022) were from the World Bank World Development Indicators database and the World Economic Outlook database.^12^, ^13^ The mortality and morbidity data (years of life lost due to premature mortality [YLL] and years lost due to disability [YLD]) were from GBD 2019 by the Institute for Health Metrics and Evaluation.^1^ For China, which collected COPD prevalence data from June, 2012, to May, 2015, under a nationally representative survey,^14^ we instead relied on these national figures, which are approximately 2·16 times higher than those from GBD 2019. Accordingly, we then scaled Chinese mortality, YLL, and YLD projections from the Institute for Health Metrics and Evaluation by the same magnitude. We then added the YLL and YLD to arrive at the disability-adjusted life-years (DALYs) associated with COPD. The total treatment cost for COPD was obtained from Dieleman and colleagues (2020),^15^ who systematically estimated national-level spending on personal health care and public health for different conditions after considering comorbidities. A more detailed data description and assumptions are shown in appendix 3 pp 7–8 and other parameter values and data sources used in the macroeconomic model are shown in appendix 3 p 8. All economic data and estimations were converted to 2017 INT$.

For 60 of 204 countries and territories, some of the necessary data were incomplete (appendix 3 pp 8–10). Similar to previous research,^10^ we used a linear projection to infer the economic burden of COPD for these countries (appendix 3 pp 10–14).

### Sensitivity analysis

We first conducted sensitivity analysis by varying the disease data for mortality and morbidity rates. We used the mean mortality and morbidity data from GBD for our baseline estimates. The best-case and worst-case estimates were calculated on the basis of the lower and upper bounds of GBD mortality and morbidity data, respectively. These bounds represent a 95% uncertainty interval for GBD estimates.^16^ We also conducted sensitivity analyses by varying the discount rates. In the main analysis, we provided estimates with a discount rate of 3%, and additional analyses calculated estimates using discount rates of 0%, 2%, 4%, and 5%. Finally, we conducted sensitivity analyses by varying model parameters within a range of 50–150% of the initial value. Results were aggregated to values with 95% uncertainty bounds by 200 random samplings from uniform distributions of the parameters. Analyses were conducted using Python version 3.9.1 software (Anaconda).

### Role of the funding source

The funders of the study had no role in study design, data collection, data analysis, data interpretation, or writing of the report.

## Results

**Figure 1:**
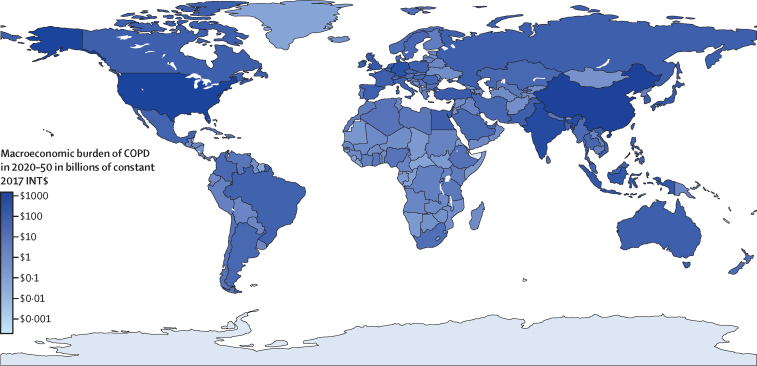
Macroeconomic burden of COPD in 2020–50 in billions of constant 2017 INT$ The darker a country's colour as displayed on the map, the higher its economic burden of COPD in billions of constant 2017 INT$. Grey areas represent countries with insufficient data. COPD=chronic obstructive pulmonary disease.

**Figure 2:**
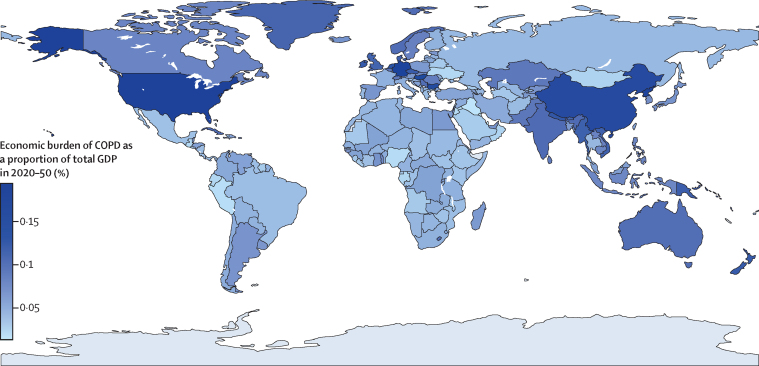
Economic burden of COPD as a proportion of total GDP in 2020–50 The darker a country's colour as displayed on the map, the higher its economic burden of COPD as a proportion of total GDP in 2020–50. Grey areas represent countries with insufficient data. COPD=chronic obstructive pulmonary disease. GDP=gross domestic product.

Total economic loss for the 144 countries with complete data and the 60 countries with missing data are shown in table 1, with the former group collectively accounting for 92·7% of the global projected population and 95·8% of the global projected GDP during 2020–50. Other discounted estimates and other estimates with randomly sampled parameters are shown in appendix 3 (pp 15–24). Of all countries, China had the largest absolute economic burden of COPD, followed by the USA and India (table 1). In terms of share of GDP, the USA, North Korea, and Germany had the largest burdens (each at around 0·19% of GDP). The per-capita burden estimates were highest in Ireland, Monaco, and the USA (table 1). The total macroeconomic burden and the burden as a proportion of GDP are shown as shaded world maps in figure 1 and figure 2.

**Table 1:** Total macroeconomic burden, economic burden as a proportion of GDP in 2020–50 adjusted for projected economic growth rate, and per capita economic burden attributable to chronic obstructive pulmonary disease mortality and morbidity in 2020–50, by country and World Bank region

	Economic loss, millions of 2017 INT$ (uncertainty interval*)	Proportion of total GDP in 2020–50, × 10^−3^ % (uncertainty interval*)	Per capita loss, 2017 INT$ (uncertainty interval*)
**East Asia and Pacific**			
American Samoa†	14 (9–20)	69 (47–98)	253 (173–364)
Australia	33 923 (26 876–41 742)	97 (77–120)	1157 (917–1424)
Brunei	261 (170–385)	40 (26–59)	549 (358–810)
Cambodia	2636 (1771–3761)	79 (53–112)	135 (91–192)
China	1 363 733 (1 033 908–1 800 765)	164 (124–216)	942 (715–1245)
Fiji	98 (49–177)	35 (18–64)	99 (50–179)
Guam†	101 (69–144)	57 (39–81)	550 (377–782)
Indonesia	96 130 (68 016–130 211)	78 (55–106)	313 (221–424)
Japan	77 318 (61 241–97 307)	69 (55–87)	662 (524–833)
Kiribati†	5 (3–8)	68 (43–101)	34 (22–51)
North Korea†	2331 (1898–2883)	190 (155–236)	88 (71–108)
South Korea	45 733 (37 163–56 162)	73 (59–89)	913 (742–1122)
Laos	1879 (1199–2840)	81 (51–122)	220 (140–333)
Malaysia	18 312 (11 597–27 465)	59 (38–89)	493 (312–739)
Marshall Islands†	5 (3–7)	77 (46–118)	72 (43–110)
Federated States of Micronesia†	9 (5–14)	97 (53–150)	68 (37–105)
Mongolia	370 (227–593)	27 (17–43)	95 (58–152)
Myanmar†	9858 (7826–11 873)	112 (89–135)	166 (132–200)
Nauru†	2 (1–3)	56 (36–83)	188 (122–278)
New Zealand	7798 (6112–9767)	120 (94–150)	1477 (1158–1850)
Northern Mariana Islands†	24 (17–34)	62 (44–89)	386 (275–554)
Palau†	8 (6–11)	127 (92–178)	428 (309–602)
Papua New Guinea†	1316 (893–1927)	115 (78–168)	113 (77–166)
Philippines	30 648 (22 824–41 651)	79 (59–107)	238 (177–323)
Samoa†	24 (16–34)	72 (49–100)	104 (70–144)
Singapore	4645 (3615–6051)	29 (23–38)	739 (575–962)
Solomon Islands†	46 (31–66)	99 (66–142)	47 (32–68)
Taiwan†	20 307 (13 686–30 213)	58 (39–86)	860 (580–1280)
Thailand	15 195 (11 280–20 778)	43 (32–59)	220 (163–300)
Timor-Leste†	103 (73–141)	88 (62–120)	61 (43–84)
Tonga†	11 (7–15)	61 (42–86)	89 (61–124)
Tuvalu†	2 (1–3)	96 (65–136)	151 (103–215)
Vanuatu†	24 (17–33)	95 (66–132)	56 (39–78)
Viet Nam	46 675 (26 482–71 176)	112 (63–170)	443 (252–676)
**Europe and central Asia**			
Albania	548 (331–834)	48 (29–73)	203 (123–309)
Andorra†	87 (65–115)	86 (63–113)	1125 (831–1481)
Armenia	736 (551–999)	58 (43–78)	252 (188–342)
Austria	7442 (6342–8827)	65 (55–77)	812 (692–964)
Azerbaijan	1412 (921–2088)	43 (28–64)	130 (85–193)
Belarus	1185 (751–2000)	32 (20–55)	130 (83–220)
Belgium	12 509 (10 127–15 222)	91 (73–110)	1044 (845–1270)
Bosnia and Herzegovina	819 (587–1135)	58 (42–80)	272 (195–377)
Bulgaria	7296 (4970–10 439)	158 (107–225)	1186 (808–1697)
Croatia	1555 (1126–2158)	48 (34–66)	415 (300–576)
Cyprus	528 (425–649)	44 (35–54)	408 (329–501)
Czech Republic	14 548 (10 473–19 143)	117 (84–154)	1363 (981–1794)
Denmark	10 024 (8000–12 376)	115 (92–142)	1655 (1321–2043)
Estonia	984 (685–1383)	66 (46–93)	788 (549–1108)
Finland	3092 (2483–3830)	48 (39–59)	557 (447–690)
France	33 624 (27 275–41 707)	49 (40–61)	502 (407–623)
Georgia	571 (432–773)	31 (23–42)	152 (115–205)
Germany	193 841 (147 414–240 605)	187 (142–232)	2352 (1789–2920)
Greece	2643 (2122–3265)	39 (32–49)	272 (218–336)
Greenland†	79 (58–105)	107 (78–143)	1406 (1030–1882)
Hungary	16 538 (10 882–23 302)	167 (110–236)	1816 (1195–2559)
Iceland	409 (300–530)	69 (51–90)	1124 (825–1457)
Ireland	37 948 (31 546–45 929)	120 (100–145)	7087 (5892–8578)
Italy	25 542 (21 622–30 103)	49 (41–58)	441 (373–520)
Kazakhstan	12 628 (8968–17 219)	89 (63–122)	588 (417–801)
Kyrgyzstan	654 (452–989)	67 (47–102)	83 (57–126)
Latvia	799 (507–1243)	48 (30–74)	481 (306–749)
Lithuania	1746 (1292–2422)	55 (40–76)	730 (540–1012)
Luxembourg	757 (599–958)	37 (29–47)	1058 (837–1340)
Moldova	462 (328–684)	45 (32–66)	123 (88–183)
Monaco†	204 (160–263)	87 (68–112)	4760 (3739–6151)
Montenegro	149 (99–213)	43 (29–62)	242 (161–346)
Netherlands	32 605 (26 048–39 900)	131 (105–161)	1877 (1500–2297)
North Macedonia	444 (288–664)	47 (31–71)	222 (144–332)
Norway	8629 (7393–9649)	99 (85–110)	1425 (1221–1593)
Poland	26 806 (20 669–35 733)	64 (50–86)	745 (575–994)
Portugal	3619 (2902–4615)	42 (34–54)	373 (299–475)
Romania†	16 274 (11 689–22 175)	85 (61–116)	915 (657–1247)
Russia	33 806 (24 630–46 311)	42 (31–58)	240 (174–328)
San Marino†	36 (26–50)	74 (53–103)	1058 (763–1475)
Serbia	3986 (2754–5691)	97 (67–139)	501 (346–716)
Slovakia	3702 (2544–5283)	75 (51–107)	700 (481–999)
Slovenia	868 (654–1167)	36 (27–48)	428 (323–576)
Spain	30 995 (25 634–37 743)	68 (56–83)	680 (562–828)
Sweden	11 949 (10 396–13 617)	82 (71–93)	1107 (963–1261)
Switzerland	13 326 (10 427–16 907)	89 (70–113)	1428 (1117–1812)
Tajikistan	1058 (706–1549)	63 (42–92)	83 (55–121)
Türkiye	48 324 (36 577–64 564)	56 (42–75)	529 (400–706)
Turkmenistan†	735 (440–1279)	33 (20–58)	104 (62–181)
Ukraine	653 (407–1013)	24 (15–37)	17 (10–26)
UK	77 553 (64 953–89 476)	108 (90–125)	1087 (910–1254)
Uzbekistan	4859 (2911–7686)	44 (27–70)	125 (75–198)
**Latin America and Caribbean**			
Antigua and Barbuda†	16 (11–23)	31 (20–45)	152 (99–220)
Argentina	13 530 (10 792–16 901)	68 (54–84)	268 (214–335)
The Bahamas	82 (51–125)	29 (18–44)	188 (116–286)
Barbados	25 (15–37)	30 (18–45)	87 (52–131)
Belize	37 (25–54)	64 (42–92)	76 (50–109)
Bolivia	1319 (820–1965)	46 (29–69)	95 (59–141)
Brazil	25 524 (21 707–30 618)	40 (34–48)	113 (97–136)
Chile	5945 (4765–7471)	51 (41–64)	300 (241–377)
Colombia	10 810 (7089–16 573)	53 (35–82)	200 (131–306)
Costa Rica	1274 (838–1896)	40 (27–60)	230 (151–342)
Cuba†	4256 (2670–5897)	102 (64–141)	391 (245–541)
Dominica†	9 (6–14)	52 (34–77)	129 (83–192)
Dominican Republic	4718 (2377–8235)	59 (30–103)	393 (198–685)
Ecuador	1037 (734–1439)	25 (18–34)	50 (35–69)
El Salvador	453 (255–724)	32 (18–52)	66 (37–106)
Grenada†	21 (15–29)	44 (32–60)	182 (130–247)
Guatemala	1061 (697–1533)	23 (15–33)	47 (31–68)
Guyana†	531 (328–800)	39 (24–58)	646 (399–974)
Haiti†	360 (208–580)	48 (27–77)	27 (16–44)
Honduras	964 (516–1614)	58 (31–97)	80 (43–134)
Jamaica	259 (152–419)	44 (26–71)	86 (50–138)
Mexico	21 175 (15 569–28 436)	37 (27–50)	147 (108–197)
Nicaragua†	380 (233–556)	40 (24–58)	49 (30–72)
Panama	1465 (1042–2039)	38 (27–53)	284 (202–395)
Paraguay	1219 (754–1778)	46 (29–67)	148 (92–216)
Peru	2204 (1591–3080)	20 (15–28)	59 (43–83)
Puerto Rico†	1403 (917–2141)	71 (47–109)	510 (333–779)
Saint Kitts and Nevis†	15 (11–22)	47 (32–67)	277 (189–392)
Saint Lucia†	40 (28–53)	75 (53–100)	214 (151–285)
Saint Vincent and the Grenadines†	15 (11–21)	42 (30–58)	137 (98–189)
Suriname	54 (33–81)	31 (19–47)	83 (52–126)
Trinidad and Tobago†	255 (142–424)	40 (22–67)	183 (102–304)
Uruguay	1214 (940–1503)	67 (52–83)	339 (262–419)
Venezuela†	4736 (2675–7633)	58 (33–93)	139 (78–224)
Virgin Islands†	63 (41–91)	53 (34–76)	647 (419–934)
**Middle East and north Africa**			
Algeria†	5340 (3837–7349)	45 (32–62)	101 (73–139)
Bahrain	343 (261–456)	17 (13–23)	165 (126–219)
Djibouti	115 (72–178)	44 (28–68)	99 (62–153)
Egypt	32 058 (19 457–46 992)	65 (39–95)	245 (149–359)
Iran†	14 450 (11 840–18 116)	53 (43–66)	152 (125–191)
Iraq	1905 (1390–2611)	16 (12–22)	34 (25–47)
Israel	8443 (6464–10 967)	70 (53–91)	792 (606–1028)
Jordan	564 (388–802)	21 (14–29)	50 (34–71)
Kuwait	949 (743–1211)	22 (17–28)	193 (151–246)
Lebanon	125 (80–183)	30 (19–44)	20 (13–29)
Libya†	4410 (2907–6276)	54 (36–77)	563 (371–801)
Malta	675 (558–814)	75 (62–90)	1528 (1263–1844)
Morocco	3351 (2352–4761)	44 (31–62)	79 (56–113)
Oman	1067 (695–1535)	26 (17–38)	174 (113–250)
Qatar	725 (540–979)	12 (9–16)	210 (157–284)
Saudi Arabia	12 497 (9234–17 259)	30 (22–42)	308 (228–425)
Syria†	797 (506–1251)	52 (33–81)	29 (18–45)
Tunisia	1216 (896–1646)	42 (31–57)	94 (69–127)
United Arab Emirates†	12 726 (7819–20 097)	70 (43–110)	1212 (744–1913)
Yemen†	1029 (670–1514)	46 (30–67)	26 (17–38)
**North America**			
Bermuda†	75 (52–106)	47 (33–66)	1274 (891–1811)
Canada	35 638 (27 170–45 095)	80 (61–101)	848 (646–1073)
USA	1 037 291 (867 830–1 174 581)	194 (162–220)	2903 (2429–3287)
**South Asia**			
Afghanistan†	633 (390–920)	41 (25–59)	12 (7–18)
Bangladesh	28 843 (25 497–42 277)	64 (56–94)	158 (140–232)
Bhutan	332 (203–551)	106 (65–175)	388 (237–643)
India	417 957 (295 685–557 049)	100 (71–133)	272 (192–362)
Maldives	164 (117–224)	59 (42–81)	300 (215–410)
Nepal	5561 (3506–8247)	125 (79–185)	165 (104–245)
Pakistan	33 366 (23 251–46 624)	90 (63–126)	118 (83–165)
Sri Lanka	7143 (4446–11 521)	86 (54–139)	325 (202–524)
**Sub-Saharan Africa**			
Angola	1388 (869–2042)	32 (20–47)	26 (16–38)
Benin	892 (559–1368)	51 (32–79)	50 (31–77)
Botswana	469 (336–649)	45 (32–62)	158 (113–219)
Burkina Faso	919 (589–1384)	47 (30–70)	29 (19–44)
Burundi	92 (56–147)	41 (25–66)	5 (3–8)
Cabo Verde	49 (33–70)	46 (31–65)	78 (52–111)
Cameroon	1627 (1039–2401)	46 (29–68)	43 (27–63)
Central African Republic†	81 (49–129)	53 (32–85)	12 (7–20)
Chad†	216 (141–318)	38 (25–56)	9 (6–13)
Comoros	37 (23–59)	47 (28–74)	32 (19–50)
Democratic Republic of the Congo	2348 (1362–3723)	57 (33–90)	17 (10–27)
Congo (Brazzaville)	119 (71–178)	38 (23–57)	15 (9–22)
Côte d'Ivoire	2826 (1822–4264)	38 (25–58)	74 (48–112)
Equatorial Guinea†	99 (60–152)	34 (21–53)	47 (28–72)
Eritrea†	126 (80–185)	43 (27–62)	27 (17–39)
Eswatini	129 (87–184)	51 (34–72)	91 (62–131)
Ethiopia	6044 (4034–8447)	34 (23–48)	38 (25–53)
Gabon	220 (151–312)	25 (17–35)	73 (50–104)
The Gambia	113 (68–179)	54 (32–85)	31 (19–50)
Ghana	4788 (2864–7270)	69 (41–105)	116 (69–176)
Guinea	1060 (645–1647)	57 (35–88)	55 (33–85)
Guinea-Bissau	93 (56–146)	67 (40–105)	34 (20–53)
Kenya	3329 (2435–4457)	36 (26–48)	46 (33–61)
Lesotho	100 (62–158)	96 (60–152)	41 (26–65)
Liberia†	59 (38–88)	32 (21–48)	8 (5–12)
Madagascar	854 (501–1359)	65 (38–103)	21 (12–34)
Malawi†	351 (233–511)	35 (23–51)	12 (8–18)
Mali	991 (563–1628)	57 (32–93)	32 (18–52)
Mauritania	294 (197–427)	33 (22–48)	44 (29–63)
Mauritius	457 (347–601)	65 (49–85)	365 (277–481)
Mozambique	605 (419–892)	39 (27–58)	13 (9–19)
Namibia	221 (146–324)	44 (29–65)	68 (45–100)
Niger	673 (412–1052)	45 (28–71)	16 (10–25)
Nigeria	5584 (3823–7627)	21 (15–29)	19 (13–26)
Rwanda	967 (600–1541)	65 (40–103)	54 (33–86)
São Tomé and Príncipe†	21 (14–30)	73 (49–106)	69 (46–99)
Senegal	1341 (893–1938)	49 (33–71)	55 (36–79)
Seychelles†	54 (40–73)	64 (48–86)	524 (390–707)
Sierra Leone	108 (67–167)	34 (21–52)	10 (6–16)
Somalia†	256 (150–428)	42 (25–71)	10 (6–17)
South Africa	8229 (6592–10 311)	48 (39–60)	120 (96–151)
South Sudan†	226 (145–337)	37 (24–55)	15 (9–22)
Sudan	1883 (1181–2850)	42 (26–63)	30 (19–46)
Tanzania	3701 (2343–5541)	48 (31–72)	40 (25–60)
Togo	585 (353–901)	77 (46–118)	50 (30–77)
Uganda	2093 (1260–3304)	48 (29–75)	31 (19–49)
Zambia	951 (613–1414)	51 (33–76)	34 (22–50)
Zimbabwe	468 (302–679)	37 (24–53)	24 (16–35)
**Others**			
Cook Islands†	8 (6–10)	88 (68–114)	462 (360–602)
Niue†	0 (0–0)	114 (83–153)	206 (149–276)
Palestine†	242 (158–359)	31 (20–45)	35 (23–52)
Tokelau†	0 (0–0)	78 (56–105)	124 (89–168)

GDP=gross domestic product.

* Uncertainty intervals in parentheses were calculated in the sensitivity analysis based on the lower and upper bounds of 95% uncertainty intervals for Global Burden of Disease Study 2019 mortality and morbidity data.

† Results imputed due to missing data.

The aggregated results for World Bank regions and country income groups are shown in table 2. Globally, we estimated the macroeconomic loss due to COPD to be INT$4·326 trillion (uncertainty interval 3·327–5·516) in 2020–50, with a discount rate of 3% in the main analysis. This estimate was INT$7·858 trillion (uncertainty interval 6·025–10·063) if undiscounted, INT$5·257 trillion (4·039–6·713) if discounted at 2%, INT$3·576 trillion (2·753–4·553) if discounted at 4%, and INT$2·970 trillion (2·289–3·776) if discounted at 5% (appendix 3 p 20). Our main result implies that the burden of COPD is equivalent to a tax of 0·111% (uncertainty interval 0·085–0·141) on cumulative global output, or to a per-capita burden of INT$490 (377–625).

**Table 2:** Total macroeconomic burden, economic burden as a proportion of total GDP, and per capita economic burden attributable to chronic obstructive pulmonary disease mortality and morbidity in 2020–50 by World Bank region, by World Bank income group, and globally

	Economic loss, billions of 2017 INT$ (uncertainty interval*)	Proportion of total GDP in 2020–50, × 10^−3^ % (uncertainty interval*)	Per capita loss, 2017 INT$ (uncertainty interval*)
**By World Bank region**			
East Asia and Pacific	1780 (1337–2358)	128 (96–170)	723 (543–958)
Europe and central Asia	712 (554–897)	87 (68–109)	765 (595–963)
Latin America and Caribbean	106 (78–145)	45 (33–61)	148 (109–202)
Middle East and north Africa	103 (71–145)	48 (33–67)	183 (126–258)
North America	1073 (895–1220)	185 (155–211)	2687 (2241–3054)
South Asia	494 (353–667)	96 (68–129)	234 (167–316)
Sub-Saharan Africa	58 (39–84)	41 (27–59)	35 (24–51)
**By World Bank country income group**			
Low income	26 (16–38)	48 (31–71)	27 (17–39)
Lower-middle income	778 (545–1066)	83 (58–114)	198 (138–271)
Upper-middle income	1633 (1230–2169)	119 (90–158)	616 (464–818)
High income	1885 (1532–2235)	123 (100–146)	1521 (1237–1804)
Global (204 countries and territories)	4326 (3327–5516)	111 (85–141)	490 (377–625)

GDP=gross domestic product.

* Uncertainty intervals in parentheses are calculated based on the lower and upper bounds of 95% uncertainty intervals for Global Burden of Disease Study 2019 mortality and morbidity data.

Among World Bank regions, the macroeconomic burden of COPD was highest in east Asia and Pacific, followed by north America, which also had the highest per-capita loss (table 2). North America also had the largest economic loss as a proportion of output, corresponding to a tax of 0·185% (uncertainty interval 0·155–0·211). East Asia and Pacific and south Asia were projected to experience the second-largest and third-largest percentage losses by 2050, respectively. As expected, the economic burden of COPD was positively associated with country income group, as were the proportions of total GDP and per-capita economic losses: high-income countries bore the greatest burdens, with a total economic loss of INT$1·885 trillion (1·532–2·235) and a per-capita loss of INT$1521 (1237–1804). By contrast, COPD was projected to cost low-income countries INT$26 billion (16–38) in total and INT$27 (17–39) per capita. The discounted estimates by World Bank region and World Bank income group at other discount rates are shown in appendix 3 (p 20).

COPD resulted in 95·0 million DALYs worldwide in 2020 (table 3). The comparison of the global distribution of economic losses with the lifetime disease burden attributable to COPD showed that the economic burden was not necessarily distributed in proportion to population size or DALYs (table 3). For example, south Asia had 25·4 million (26·8% of the global total) COPD DALYs in 2020, which was broadly in line with its population of 1856 million people (23·9% of the global total); however, the economic loss was only INT$494 billion (11·4% of the global total). By contrast, with a population of 369 million (4·7% of the global total), north America had 5·4 million (5·7% of the global total) COPD DALYs in 2020, but an economic loss of INT$1073 billion (24·8% of the global total). East Asia and Pacific had both a large health burden and a large economic burden, with 47·4 million (49·9% of the global total) COPD DALYs, an economic loss of INT$1780 billion (41·1% of the global total), and a population of 2380 million (only 30·6% of the global total). Overall, although LMICs had an economic loss of INT$2437 billion (56·4% of the global total) due to COPD, their disease burden measured in DALYs reached 82·7 million (87·1% of the global total; table 3).

**Table 3:** Comparison of macroeconomic burden and lifetime health burden of chronic obstructive pulmonary disease, by World Bank region and country income group

	**Economic burden in 2020–50, billions of 2017 INT$ (global %)**	**DALYs in 2020, millions (global %)**	**Total GDP in 2020, billions of 2017 INT$ (global %)**	**Population in 2020, millions (global %)**
**By World Bank region**				
East Asia and Pacific	1780 (41·1%)	47·4 (49·9%)	39 654 (32·2%)	2380 (30·6%)
Europe and central Asia	712 (16·4%)	7·7 (8·1%)	30 836 (25·0%)	924 (11·9%)
Latin America and Caribbean	106 (2·5%)	3·5 (3·7%)	9046 (7·3%)	652 (8·4%)
Middle East and north Africa	103 (2·4%)	1·8 (1·9%)	6965 (5·7%)	459 (5·9%)
North America	1073 (24·8%)	5·4 (5·7%)	21 616 (17·6%)	369 (4·7%)
South Asia	494 (11·4%)	25·4 (26·8%)	10 796 (8·8%)	1856 (23·9%)
Sub-Saharan Africa	58 (1·3%)	3·6 (3·8%)	4191 (3·4%)	1136 (14·6%)
**By World Bank country income group**				
Low income	26 (0·6%)	2·8 (2·9%)	1103 (0·9%)	665 (8·6%)
Lower-middle income	778 (18·0%)	33·3 (35·0%)	22 506 (18·3%)	3324 (42·8%)
Upper-middle income	1633 (37·8%)	46·6 (49·0%)	42 950 (34·9%)	2555 (32·9%)
High income	1884 (43·6%)	12·2 (12·8%)	56 546 (45·9%)	1204 (15·5%)
Global (204 countries and territories)	4326 (100%)	95·0 (100%)	123 104 (100%)	7782 (100%)

DALY=disability-adjusted life-year. GDP=gross domestic product.

Our results show that the effects of treatment costs play a more important role in high-income countries than in low-income countries. In high-income countries, physical capital loss due to the payment of treatment costs accounts for 29% of the global economic burden due to COPD, decreasing to 21% in upper-middle-income countries and 9% in lower-middle-income and low-income countries (appendix 3 p 25). Across regions, treatment costs play the largest role in Europe and central Asia, with 28% of the total economic burden attributed to physical capital loss, and the smallest role in south Asia and sub-Saharan Africa at 8% (appendix 3 p 26).

## Discussion

To our knowledge, this study is the first to estimate the global economic costs of COPD using a method that accounts for economic adjustment mechanisms applied consistently across a set of 204 countries and territories. Our findings fill several knowledge gaps. First, this study shows that between 2020 and 2050, COPD will cost the world economy INT$4·3 trillion, which is nearly half of the aggregate GDP of India (the world's third-largest economy in constant 2017 INT$) in 2019. According to Organisation for Economic Co-operation and Development data, official development assistance from official donors was INT$155·9 billion in 2019,^17^ which implies that the savings from eliminating the macroeconomic burden of COPD during 2020–50 would cover almost 30 years of official development assistance. Second, for the first time, this study estimated the macroeconomic burden for all countries in the world using a rigorous approach that accounts for economic adjustment mechanisms and reflects the fact that health-care expenditures would otherwise have been used for savings or investment. Third, our study shows that the health and economic burdens of COPD are distributed unequally across countries and regions.

Despite the high DALY burden and despite being home to roughly 85% of the global population, LMICs only account for 56·4% of COPD's global economic burden. Higher levels of education in the workforce can explain the disproportionately high economic toll of COPD in high-income countries, implying that for a given number of DALYs due to COPD, resulting human capital reductions will be more pronounced the more educated the workforce. Furthermore, the more advanced health systems (eg, in terms of COPD diagnosis, treatment, and rehabilitation capabilities) found in high-income countries imply higher treatment costs and, thus, greater reductions in savings. The economic burden of COPD in low-income countries is likely to rise if emerging markets for tobacco companies continue to grow without regulation, the number of people exposed to air pollution increases due to urbanisation, and the epidemiological transition from infectious diseases to non-communicable diseases progresses as life expectancy in LMICs increases.^4^

Across regions, east Asia and Pacific faces the largest economic toll from COPD, followed by north America and Europe and central Asia. These three regions account for 82·4% of the global economic cost of COPD. This is partly due to the large economic burdens of COPD in China and the USA: our findings showed that China has the largest absolute economic burden, and the USA has the largest economic burden of COPD in terms of the proportion of GDP. For China, the high economic burden of COPD is largely attributed to its huge health burden. A nationally representative survey estimated that among the population aged 20 years and older, the overall prevalence of spirometry-defined COPD was 8·6%.^14^ A high prevalence of smoking and severe air pollution largely drive the substantial COPD burden in China. With respect to smoking, China is the world's largest producer and consumer of tobacco, and has the highest mortality attributed to tobacco.^18^ In 2018, tobacco was used by 308 million (27%) of the population aged 15 years and older in China, a figure that reached as high as 50% among adult men. More than 700 million people in China are exposed to secondhand smoke.^19^ Severe air pollution is also partly responsible for China's high COPD burden,^20^ leading to 39% of total COPD deaths.^1^

Given the substantial consequences of COPD for the global economy and for population health and wellbeing, our findings highlight that investing in effective public health interventions to reduce the COPD burden is essential. Countries should reduce smoking, air pollution, and other risk factors for COPD; launch educational campaigns and strengthen health education components in clinical, workplace, and community settings to increase the knowledge and awareness of COPD; invest in COPD screening equipment; and invest in more research to identify cost-effective population health interventions to prevent or treat COPD.^21^ With respect to investing in screening equipment, spirometry pulmonary function test equipment is currently unavailable in most clinics and hospitals in many LMICs. For example, Malawi, a country of 19·3 million people, has had only one spirometer for clinical diagnostic purposes for many years.^4^ A scarcity of testing equipment results in a substantial unmet need for COPD treatment—most patients with COPD are asymptomatic and unaware of their disease status but need COPD care. In China, only 2·6% of patients with spirometry-defined COPD were aware of their condition.^14^ Screening and identifying COPD early can prevent disease progression and reduce health and economic burdens.

More research into cost-effective interventions is needed. For example, the effectiveness of community-based COPD screening is underexplored in some countries, even though such programmes have shown promising results with low costs.^22^ As a recent systematic review showed, there is “no direct evidence available to determine the benefits and harms of screening asymptomatic adults for COPD”.^23^ Because of the current scarcity of evidence on community-based COPD screening, not a single country worldwide has adopted such a programme at scale, and major guidelines (eg, by the Global Initiative for Chronic Obstructive Lung Disease and the US Preventive Services Task Force) generally suggest conducting pulmonary function tests only for symptomatic patients.^24^ However, this passive early detection strategy is likely to lead to underdiagnosis of COPD due to the latency, non-specificity, and heterogeneity of early COPD symptoms.^25^ For example, in China, 60% of patients with COPD diagnosed by a pulmonary function test are asymptomatic^14^ and, thus, would not be reached by passive early detection strategies, which require patients to self-detect early COPD symptoms and actively seek diagnosis and care in the health-care system. Additional practical evidence is needed to establish the effectiveness of active community-based screening for COPD and thus reduce the COPD morbidity and mortality rate going forward.

Our approach has several limitations (appendix 3 p 27). First, we relied on the extrapolation of COPD-related health expenditures for all countries except for the USA, under the assumption that per-case costs are proportional to per-capita health expenditure. Although this could lead to an underestimation or overestimation of country-specific treatment costs for COPD, this technique is widely used in other studies on the macroeconomic burden of diseases.^10^, ^26^, ^27^ Second, we had to impute the economic burden of COPD for a subset of 60 of 204 countries and territories via linear regression. With the imputed countries accounting for only 7% of the global population and 4% of global GDP, this should not substantially affect our results. Third, we did not include behavioural changes—such as changing labour force participation—among family members who might need to care for patients with COPD. Thus, along this dimension, our findings provide a lower bound for the economic costs of COPD because we did not account for the full costs of patient care.^28^, ^29^

In conclusion, the global economic burden of COPD is substantial. Our study stresses the urgent need to invest in global efforts to curb COPD and its associated health and economic burdens. Investments in effective interventions against COPD do not represent a burden but could instead provide substantial economic returns in the foreseeable future.

## Data sharing

No individual-level data were used in this modelling study. Data from this modelling study are available with publication. The data are available to anyone who requests them for any non-commercial purposes. The data can be accessed by contacting SC (simiao.chen@uni-heidelberg.de), who will provide guidance on how to use and interpret the data.

## Declaration of interests

We declare no competing interests.

## Supplementary Materials

Chinese translation of the abstract

German translation of the abstract
