# Supplementary material for: The global economic burden of chronic obstructive pulmonary disease for 204 countries and territories in 2020–50: a health-augmented macroeconomic modelling study
Source: Lancet Glob Health. 2023 Jul 18;11(8):e1183–93. doi: 10.1016/S2214-109X(23)00217-6 (PMC10369014; doi:10.1016/S2214-109X(23)00217-6)
Supplement: Chinese translation of the abstract [file mmc1.pdf]

THE LANCET
Global Health

Supplementary appendix 1

This translation in Chinese was submitted by the authors and we reproduce it as supplied. It has not been peer reviewed. *The Lancet*'s editorial processes have only been applied to the original in English, which should serve as reference for this manuscript.

此简体中文译文由作者提交，我方按照提供的版本刊登。此译文并未经过同行审阅。医学期刊《柳叶刀》的编辑流程仅适用于英文原稿，英文原稿应作为此手稿的参考。

Supplement to: Chen S, Kuhn M, Prettner K, et al. The global economic burden of chronic obstructive pulmonary disease for 204 countries and territories in 2020–50: a health-augmented macroeconomic modelling study. *Lancet Glob Health* 2023; **11:** e1183–93.

Chinese

摘要

背景：慢性阻塞性肺疾病（简称“慢阻肺病”）是全球第三大疾病死因，同时造成了巨大的经济负担。深入了解慢阻肺病的经济影响是制定合理的、以证据为基础的政策的重要前提。我们研究旨在估计慢阻肺病的全球宏观经济损失并调查其在各国间的分布。

方法：我们根据健康增益宏观经济模型计算了 204 个国家和地区的由慢阻肺病导致的宏观经济损失。该模型考虑了（1）慢阻肺病死亡和患病对劳动力供给的影响；（2）不同性别、不同年龄的慢阻肺病患者教育和工作经历的差异；（3）慢阻肺病治疗费用对物质资本积累的影响。我们从 2019 年全球疾病负担研究、世界银行数据库和文献在内的多个公共数据源获取了数据。我们通过比较 2020 年至 2050 年间基于当前疾病患病率进行预测的情景和没有慢阻肺病患病率的反事实情景，以国内生产总值（GDP）为指标，计算了慢阻肺病的宏观经济损失。

结果：2020-2050 年间，全球慢阻肺病的宏观经济负担为 4.326 万亿国际美元（不确定性区间 3.327-5.516，以 2017 年不变价格计），相当于慢阻肺病每年给世界经济造成 0.111%（不确定性区间 0.085-0.141）的额外税收负担。中国和美国面临着世界上最大的慢阻肺病经济负担，分别为 1.363 万亿国际美元（不确定性区间 1.034-1.801）和 1.037 万亿国际美元（不确定性区间 0.868-1.175）。

结论：慢阻肺病给宏观经济带来了巨大的负担，这种负担在不同的国家、地区和收入水平的人群之间分布不均。我们的研究强调，迫切需要投资于全球减轻慢阻肺病的行动，以减少其所致健康和经济负担。投资于针对慢阻肺病的有效干预措施不应被视作一种负担，而应当意识到，这是一种可以在可预见的未来带来可观经济回报的投资。
