# Supplementary material for: The global economic burden of chronic obstructive pulmonary disease for 204 countries and territories in 2020–50: a health-augmented macroeconomic modelling study
Source: Lancet Glob Health. 2023 Jul 18;11(8):e1183–93. doi: 10.1016/S2214-109X(23)00217-6 (PMC10369014; doi:10.1016/S2214-109X(23)00217-6)
Supplement: German translation of the abstract [file mmc2.pdf]

THE LANCET
Global Health

Supplementary appendix 2

This translation in German was submitted by the authors and we reproduce it as supplied. It has not been peer reviewed. *The Lancet*'s editorial processes have only been applied to the original in English, which should serve as reference for this manuscript.

Diese Übersetzung in deutscher Sprache wurde von den Autoren eingereicht und wir reproduzieren sie wie vorgelegt. Die Übersetzung wurde nicht von Experten begutachtet. Die redaktionellen Prozesse von The Lancet wurden nur auf das Original in englischer Sprache angewendet, das als Referenz für dieses Manuskript dienen soll.

Supplement to: Chen S, Kuhn M, Prettner K, et al. The global economic burden of chronic obstructive pulmonary disease for 204 countries and territories in 2020–50: a health-augmented macroeconomic modelling study. *Lancet Glob Health* 2023; **11:** e1183–93.

German

Abstract

Hintergrund: Die chronisch obstruktive Lungenerkrankung (COPD) ist weltweit die dritthäufigste Todesursache und stellt eine erhebliche wirtschaftliche Belastung dar. Ein umfassendes Verständnis der ökonomischen Auswirkungen von COPD ist eine wichtige Voraussetzung für eine fundierte, evidenzbasierte Politikgestaltung. Zielsetzung dieser Studie war die makroökonomische Belastung von COPD für jedes Land zu schätzen und ihre Verteilung über die Weltregionen zu ermitteln.

Methodik: Wir haben die wirtschaftliche Belastung durch COPD für 204 Länder und Gebiete auf Grundlage eines makroökonomischen Modells berechnet, das (i) die Folgen von COPD Mortalität und Morbidität für das Arbeitskräfteangebot, (ii) alters- und geschlechtsspezifische Unterschiede bezüglich Ausbildung und Berufserfahrung der von COPD betroffenen Personen und (iii) die Auswirkungen von COPD Behandlungskosten auf die Akkumulation von physischem Kapital berücksichtigt. Wir haben Daten aus verschiedenen öffentlichen Quellen wie der Global Burden of Disease Study 2019, der Datenbank der Weltbank sowie der makroökonomischen Literatur verwendet. Die makroökonomische Belastung von COPD wurde als Differenz ermittelt zwischen dem Bruttoinlandsprodukt (BIP) für ein Status-quo Szenario, in dem die Krankheitsprävalenz auf der Grundlage aktueller Schätzungen prognostiziert wurde, und einem kontrafaktischen Szenario, in dem die COPD-Prävalenz von 2020 bis 2050 auf Null gesetzt wurde.

Ergebnisse: Unseren Ergebnissen zufolge wird COPD die Weltwirtschaft von 2020 bis 2050 INT$4,326 Billionen (Min-Max-Spanne 3,327-5,516; zu konstanten Preisen von 2017) kosten. Dieser ökonomische Effekt entspricht einer Steuer von 0.111% (0.085-0.141) auf das kumulierte weltweite Bruttoinlandsprodukt. China und die USA tragen die jeweils größte wirtschaftliche Belastung durch COPD, die sich auf INT$1,363 Billionen (Min-Max-Spanne 1,034-1,801) in China bzw. INT$1,037 Billionen (Min-Max-Spanne 0,868-1,175) in den USA beläuft.

Schlussfolgerung: Die makroökonomische Belastung durch COPD ist beträchtlich und ungleich auf die einzelnen Länder, Weltregionen und Einkommensstrata verteilt. Unsere Studie zeigt, wie dringlich globale Anstrengungen zur Eindämmung der gesundheitlichen und wirtschaftlichen Belastung durch COPD sind. Wirksame Interventionen gegen COPD sollten nicht als Kosten betrachtet werden, sondern als Investitionen, die in absehbarer Zeit erhebliche wirtschaftliche Erträge erbringen können.
