# Supplementary material for: The global economic burden of chronic obstructive pulmonary disease for 204 countries and territories in 2020–50: a health-augmented macroeconomic modelling study
Source: Lancet Glob Health. 2023 Jul 18;11(8):e1183–93. doi: 10.1016/S2214-109X(23)00217-6 (PMC10369014; doi:10.1016/S2214-109X(23)00217-6)
Supplement: Supplementary appendix 3 [file mmc3.pdf]

THE LANCET
Global Health

Supplementary appendix 3

This appendix formed part of the original submission and has been peer reviewed. We post it as supplied by the authors.

Supplement to: Chen S, Kuhn M, Prettner K, et al. The global economic burden of chronic obstructive pulmonary disease for 204 countries and territories in 2020–50: a health-augmented macroeconomic modelling study. *Lancet Glob Health* 2023; **11:** e1183–93.

Appendix file

Contents

A: Health burden of chronic obstructive pulmonary disease 2
B: Modeling details 3
C: Data description 7
D: Imputation 10
E: Discounted estimates 14
F: Sensitivity analysis on parameters 20
G: Contribution of treatment costs 24
H: Strengths and limitations 26
I: CHEER guideline 27

In this supporting information appendix to the paper "The global economic burden of chronic obstructive pulmonary disease for 204 countries and territories in 2020–2050: a health-augmented macroeconomic modeling study," we provide additional details related to our study, including the mathematical formulation of our model and detailed data sources.

A: Health burden of chronic obstructive pulmonary disease

Figures S1–S3 show the health burden of chronic obstructive pulmonary disease (COPD). The numbers are based on the Global Burden of Disease Study (2020).[1]

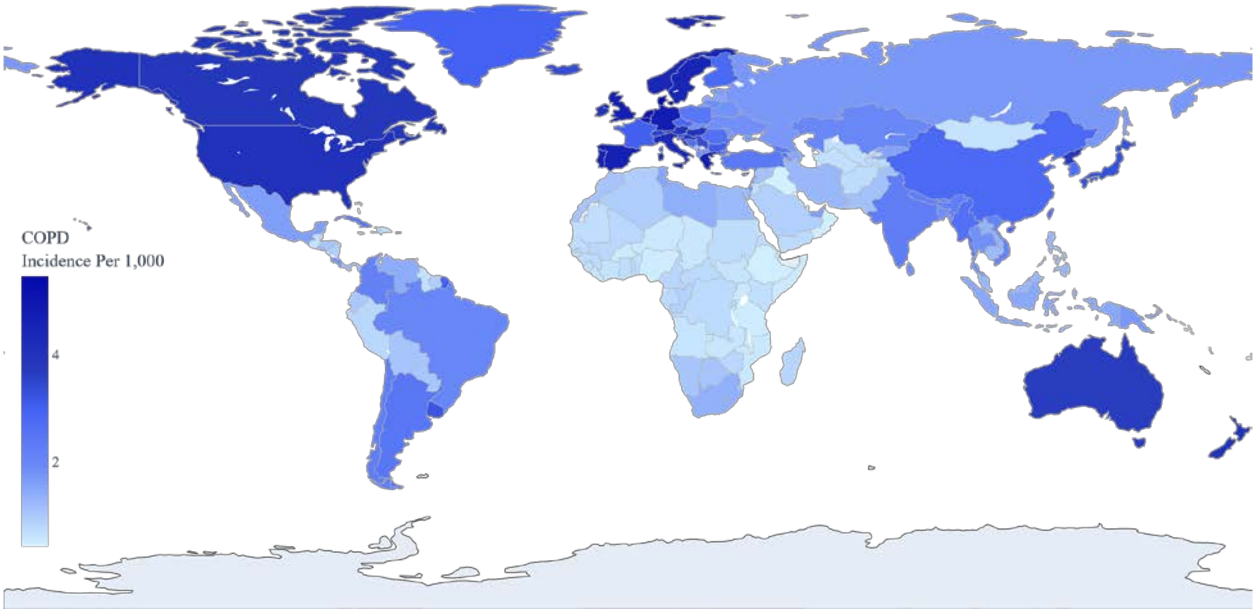

*Gray areas represent countries with insufficient data

Figure S1. COPD incidence rate (per 1,000) in 2019

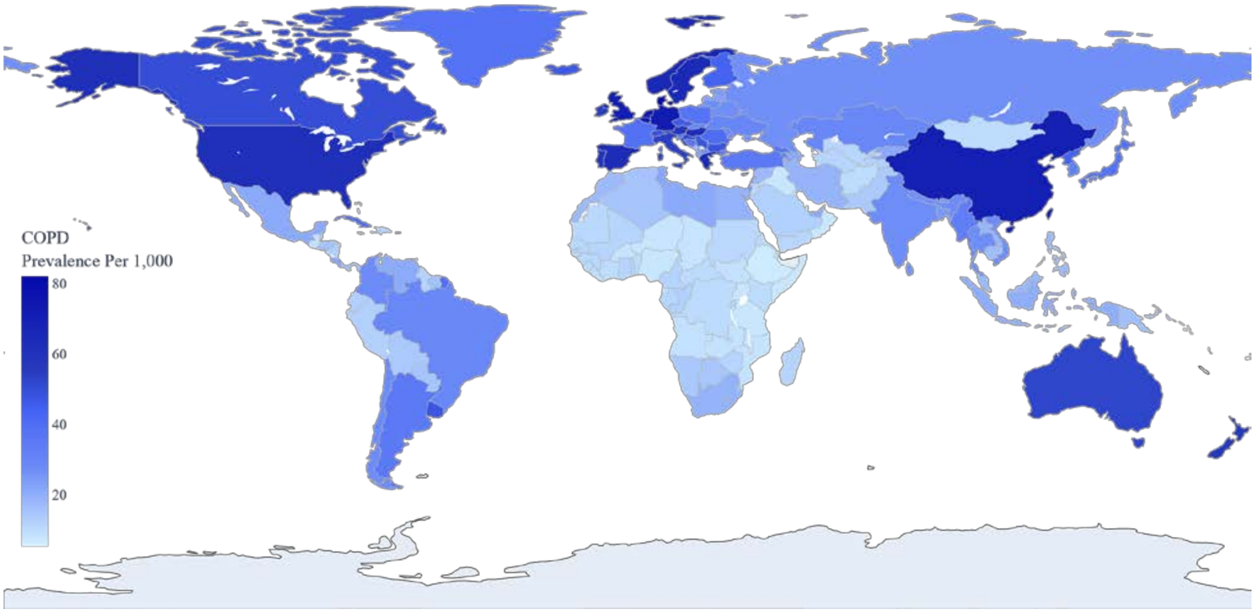

Figure S2. COPD prevalence (per 1,000) in 2019

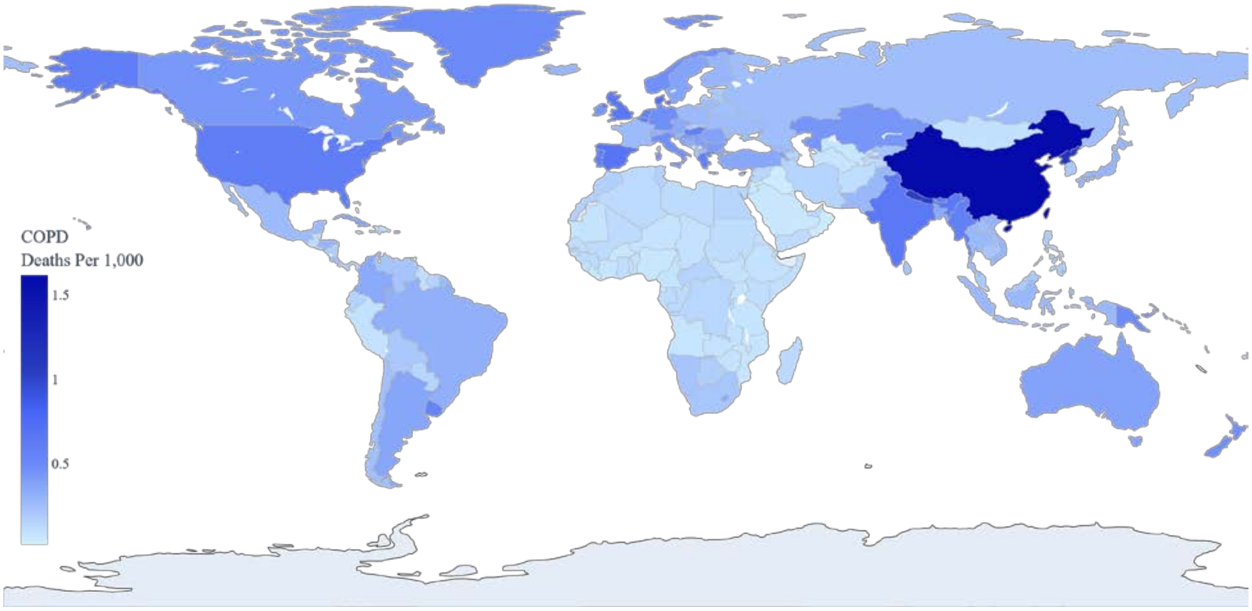

Figure S3. COPD mortality rate (per 1,000) in 2019

B: Modeling details

This detailed model description follows our previous contributions, wherein we applied the framework to estimate the economic burden of noncommunicable diseases in China, Japan, and South Korea[2] and in the United States and European countries;[3,4] the burden of road injuries;[5] the burden of cancer;[6] and the burden of risk factors such as tobacco[7] and air pollution.[8]

We aimed to quantify COPD's impact on economic output through healthcare expenditures and through productivity losses due to mortality and morbidity. For each country, we did the following analysis:

Step 1. We identified the disease burden of COPD (in terms of mortality, morbidity, and treatment costs).
Step 2. We constructed economic projections for two scenarios: (i) the aggregate output Y_t in the status quo scenario without additional interventions to reduce COPD mortality and morbidity in year *t* and (ii) the aggregate output $\overline{Y_t}$ in the counterfactual scenario with complete COPD elimination at zero cost in year *t*. The economic projections utilize a macroeconomic production function and can be further decomposed into two parts:

a) Projections of effective labor supply and
b) Projections of physical capital accumulation.

Step 3. We calculated the economic loss as the cumulative difference in projected annual gross domestic product (GDP) between these two scenarios:

$$\Delta Y = \sum_{t=2020}^{2050} (\bar{Y}_t - Y_t)$$

Production function
Consider an economy in which time $t = 1,2, \ldots, \infty$ evolves discretely. Building upon Lucas (1988),[9] we considered the following production function for this economy:

$$Y_t = A_t K_t^{\alpha} H_t^{1-\alpha}, \tag{1}$$

where Y_t is aggregate output; A_t is the technological level at time t, which we assume evolves exogenously; K_t is the physical capital stock (i.e., machines, factory buildings, etc.); and H_t represents aggregate human capital. The parameter α is the elasticity of final output with respect to physical capital. Solow's framework, upon which the World Health Organization's original EPIC macroeconomic model is based,[10] only considers physical capital and *raw labor* as factors of output production.[11] However, the aggregate production function recognizes that output is not only produced with these factors but also with *effective labor*, including health, which is a crucial determinant.

Physical capital evolves according to

$$K_{t+1} = (1-\delta)K_t + Y_t - C_t - TC_t = (1-\delta)K_t + s_t Y_t \,, \tag{2}$$

where δ refers to the depreciation rate, s_t refers to the saving rate, TC_t refers to the costs of ongoing treatment of COPD, and C_t refers to the amount of consumption. From Equation (2), it follows that the saving rate is defined as

$$s_t = 1 - \frac{C_t + TC_t}{Y_t}.$$

Note that aggregate output Y_t is used for three purposes: (i) to pay treatment costs TC_t (hospitalization, medication, etc.), (ii) to consume the amount C_t, and (iii) to save.

Individuals of age group a are endowed with $h_t^{(a)}$ units of human capital and supply $\ell_t^{(a)}$ units of labor from age 15 up to their retirement at age R, i.e., for $a \in [15, \mathrm{R}]$. Children younger than 15 and retirees older than R do not work. R varies by country and could correspond to a high age (e.g., some people older than 80 could also be working). In the theoretical derivations, R indicates the upper bound of the summation. In our simulations, we used labor projections data from the International Labour Organization where positive values for the labor force exist for cohorts older than 65. Aggregate human capital in the production function (1) is then defined as the sum over the age-specific effective labor supply of each age group:

$$H_t = \sum_{a=15}^{R} h_t^{(a)} \ell_t^{(a)} N_t^{(a)} \;, \tag{3}$$

where N_t^a denotes the number of individuals in age group a. Note that aggregate human capital increases with the number of working-age individuals who live in the economy (i.e., with a higher $N_t = \sum_{a=15}^{R} N_t^{(a)}$), with individual

human capital endowment (i.e., with a higher $h_t^{(a)}$ for at least one a), and with labor supply (i.e., with a higher $\ell_t^{(a)}$ for at least one a). This human capital function itself allows for the substitution of capital (machines or robots) for workers. In addition, if the disease predominantly affects one age group, then workers from other age groups can substitute.

We followed Mincer (1974)[12] and constructed the average human capital of the cohort aged a according to an exponential function of education and work experience:

$$h_t^{(a)} = \exp\left[\eta_1\left(ys_t^{(a)}\right) + \eta_2\left(a - ys_t^{(a)} - 5\right) + \eta_3\left(a - ys_t^{(a)} - 5\right)^2\right], \tag{4}$$

where η_1 is the semi-elasticity of human capital with respect to average years of education as given by $ys_t^{(a)}$, and η_2 and η_3 are the semi-elasticities of human capital with respect to experience of the workforce $\left(a - ys_t^{(a)} - 5\right)$ and experience of the workforce squared $\left(a - ys_t^{(a)} - 5\right)^2$, respectively. Here, we assumed a school entry age of 5 years throughout.

Impact of COPD on labor supply
Following Bloom et al. (2020)[2] and Chen et al. (2018, 2019a, 2023, 2019b, 2019c),[3,5-8] the evolution of labor supply in the status quo scenario is given by

$$L_t^{(a)} = \ell_t^{(a)} N_t^{(a)} \text{ with } N_t^{(a)} = \left[1 - \sigma_{t-1}^{(a-1)}\right] N_{t-1}^{(a-1)}, \tag{5}$$

where $\sigma_t^{(a)}$ is the overall mortality rate of age group a at time t. Mortality and morbidity reduce effective labor supply. The reduction of the population size $N_t^{(a)}$ captures the mortality effect.

Let $\sigma_{r,t}^{(a)}$ denote the mortality rate of people in age group a due to COPD and let $\sigma_{-r,t}^{(a)}$ be the overall mortality rate due to causes other than COPD, then we have

$$\left(1 - \sigma_t^{(a)}\right) = (1 - \sigma_{r,t}^{(a)})(1 - \sigma_{-r,t}^{(a)}).$$

Next, we considered the mortality effect of COPD. In general, it reduces labor supply by reducing the population $N_t^{(a)}$ (through $\sigma_{r,t}^{(a)}$). In the counterfactual case, where COPD is eliminated from time $t = 0$ onward, the evolution of labor supply is defined similarly to Equation (5), but with a different overall mortality rate ($\sigma_{-r,t}^{(a)}$ instead of $\sigma_t^{(a)}$). For simplicity, we assumed that the number of births is the same in both cases at each point in time t.

In the counterfactual scenario, in which we denote variables with an overbar, the size of the cohort aged a at time t ($\bar{N}_t^{(a)}$) evolves according to

$$\bar{N}_t^{(a)} = \left[1 - \sigma_{-r,t-1}^{(a-1)}\right] \bar{N}_{t-1}^{(a-1)}, \bar{N}_0^{(a)} = N_0^{(a)}, \bar{N}_t^{(0)} = N_t^{(0)},$$

Following Bloom et al. (2020),[2] the loss of labor due to mortality accumulates over the years according to

$$\bar{N}_t^{(a)} = N_t^{(a)} / \prod_{\tau=0}^{\min\{t,a\}-1} \left[1 - \sigma_{r,t-1-\tau}^{(a-1-\tau)}\right].$$

The reduction of the labor participation rate $\ell_t^{(a)}$ captures the morbidity effect because people with an illness typically reduce their labor supply, either by reducing working hours or by leaving the workforce. Following Bloom et al. (2020)[2], the labor participation rate in the counterfactual scenario $\bar{\ell}_t^{(a)}$ can be calculated as

$$\bar{\ell}_t^{(a)} \approx \ell_t^{(a)} / \prod_{\tau=0}^{\min\{t,a\}-1} \left[1 - p^{\tau} \sigma_{r,t-1-\tau}^{(a-1-\tau)} \xi^{(a-1-\tau)}\right],$$

where $\xi^{(a)}$ measures the size of the morbidity effect relative to the relevant mortality rate and where p^{τ} is the probability that a patient died from COPD by time t.

Because the impact of morbidity is hard to estimate directly, we first defined

$$\xi^{(a)} = \frac{\mathit{loss\ of\ labor\ due\ to\ morbidity\ in\ age\ group}\ \mathrm{a}}{\mathit{loss\ of\ labor\ due\ to\ mortality\ in\ age\ group}\ \mathrm{a}}\ . \tag{6}$$

Next, we assumed that the following holds in any given year for each age group a:

$$\xi^{(a)} = \frac{YLD^{(a)}}{YLL^{(a)}}\ , \tag{7}$$

where $YLD^{(a)}$ represents the years lived with COPD and $YLL^{(a)}$ represents the years of life lost due to COPD. Notice that $\xi^{(a)}$ can be calculated from the corresponding disability-adjusted life year data reported by the Global Burden of Disease Study (2020).[1]

In sum, by reducing the prevalence of COPD, the *counterfactual scenario* is associated with an increase in labor supply as compared with the *status quo scenario.* We approximated the change in labor supply (at time t for age group a) by

$$\Delta L_t^{(a)} \approx \ell_t^{(a)} N_t^{(a)} \sum_{\tau=0}^{\min\{t,a\}-1} \sigma_{r,t-1-\tau}^{(a-1-\tau)} \left[1 + p^{\tau} \xi^{(a-1-\tau)}\right]. \tag{8}$$

Bloom et al. (2020)[2] provide the detailed mathematical proof.

Impact of COPD on physical capital accumulation

COPD also impedes physical capital accumulation because savings finance part of the treatment costs. Following Bloom et al. (2020)[2] and Chen et al. (2018),[3] physical capital accumulation in the counterfactual scenario can be written as

$$\bar{K}_{t+1} = \bar{s}_t \bar{Y}_t + (1-\delta)\bar{K}_t\ , \tag{9}$$

$$\bar{s}_t \bar{Y}_t = \bar{I}_t = \bar{Y}_t - \bar{C}_t = \mathrm{s}_t \bar{Y}_t + \chi T C_t, \tag{10}$$

where χ is the fraction of the treatment cost that is diverted to savings. The counterfactual saving rate is thus defined by

$$\bar{s}_t = \frac{s_t \bar{Y}_t + \chi T C_t}{\bar{Y}_t}.$$

For more details, see Bloom et al. (2020)[2] and Chen et al. (2018).[3]

Because COPD is assumed to be eliminated in the counterfactual scenario, the resources that were devoted to its treatment can now be used for savings or for consumption. Notice that this creates an income effect that, in reality, could affect the division of households' income between savings and consumption. For tractability, we assume that aggregate investment consists of two parts in the counterfactual scenario: a fixed share s_t of total output and an additional part from TC_t that would otherwise have been used to pay to treat COPD:

$$\bar{I}_t = s_t \bar{Y}_t + \chi TC_{t.}$$

Similarly, for the case of a partial reduction in COPD prevalence by ρ, we have

$$\bar{I}_t = s_t \bar{Y}_t + \rho \chi TC_t.$$

The intuition is that if COPD is partially eliminated, the treatment cost that is diverted to savings should be added back proportionally.

C: Data description

Education
Age-specific educational attainment data were obtained from the Barro-Lee Educational Attainment Database,[13] which provides educational attainment data in five-year age groups up to 2010. For 2010–2030, no age-specific data are available, but the database provides projections for the population aged 15–64. We approximated the age-specific estimates by assuming that educational attainment for each age group grows at the same rate. Because the Barro-Lee database presents data in five-year intervals, linear interpolation was adopted to extend the estimates for each year. For 2030–2050, we projected educational attainment by assuming the same growth rate as in 2010–2030 for each age and sex group.

Mortality/morbidity
Mortality and morbidity (measured in YLLs and YLDs) due to COPD up to 2019 were obtained from the recently updated Global Burden of Disease (GBD) estimates.[1] To extend the estimates beyond 2019, we assumed that the mortality rates from COPD grow at the same rate as in 2010–2019 for each country. Morbidity estimates were obtained similarly. If the projected mortality rate grew too large (i.e., if it more than doubled the current annual rate in 30 years), we limited the mortality rate's growth rate to 2%.

GDP projection
The GDP estimates (in constant 2017 international dollars or INT$) up to 2020 are from the World Bank database.[14] The GDP growth rates for 2021–2027 are from the International Monetary Fund's World Economic Outlook as of April 2022.[15] We assumed that growth beyond 2027 will be the same as in 2015–2019.

Physical capital
For each country, the physical capital stock (in 2017 INT$) was obtained from the Penn World Table projections (2021),[16] with the value for the output elasticity of physical capital (the percentage change in output for a 1% change in the physical capital stock) following standard economic estimates.[17]

Labor and population projection
For each country, the labor participation rate and population estimates (by five-year age group) were obtained from the International Labour Organization database for 2015–2030.[18] For estimates beyond 2030, we assumed that growth of the labor participation rate remains the same as in 2020–2030.

Saving rate and health expenditure
We obtained country-specific saving rates and health expenditures from the World Bank database (2020b).[19] For the projection, we assumed that the saving rates remain constant (at the average in 2010–2019), while health expenditures (as a percentage of GDP) grow at the same rate as in 2000–2019.

Treatment costs
Total treatment cost for COPD in the United States is based on Dieleman et al. (2020).[20] Their total treatment cost estimate includes inpatient and outpatient medical costs due to COPD, which amount to INT$35.0 billion (34.3 billion in U.S. dollars) in 2016 (1.268% of health expenditures for all disease categories). We calculated the per case costs for the countries with data and extrapolated costs for countries without data, under the assumption that per case costs are proportional to per capita health expenditure, as was done in previous studies.[5,21,22] Specifically, we extrapolated COPD treatment cost per case using GBD disease prevalence data and extrapolated costs to other countries using a scaling factor (defined as the ratio of health expenditures per capita between the country of interest and the country

with available data). We then calculated COPD treatment cost per capita by multiplying treatment cost per case by COPD prevalence. For years after 2010, we assumed that the COPD treatment cost would grow at the same rate as per capita health expenditure for each country. To make estimates among countries comparable, we convert all costs to the base year of 2017.

Discount rate

The discount rate of 3% is somewhat standard in global health and is recommended, for example, by the Panels on Cost-effectiveness in Health and Medicine.[23] However, an appropriate discount rate depends on the current economic context and varies across countries. For example, the discount rate for low- and middle-income countries should be larger than for high-income countries, e.g., 5%.[24] For country-specific discount rates, 17 of 22 national guidelines for economic evaluations recommend discount rates ranging from 1.5% to 5%.[25] Therefore, we have chosen 3% as the discount in the main analysis, and we provide the projected economic burden if discounted at 0%, 2%, 4%, and 5% in the Appendix.

Other parameter values and data sources

The dynamics of individual human capital are based on a Mincerian specification of the dependence of individual productivity on education and experience and the corresponding returns.[12] The estimated parameters for the Mincerian specification come from Psacharopoulos and Patrinos (2018)[26] for education and from Heckman et al. (2006)[27] for experience.

Table S1 shows the parameter values used in the model and their data sources, where definitions for parameters are consistent with Bloom et al. (2020)[2] and Chen et al. (2019a, 2023, 2019b).[5-7]

Table S1. Parameter values and data sources

Parameter	Definition	Value	Source
α	Capital share	Country specific	Penn World Table (2021)[16]
δ	Depreciation rate	0.05	Grossmann et al. (2013)[28]
η_1	Mincer elasticity of education	0.091	Psacharopoulos and Patrinos (2018)[26]
η_2	First-degree Mincer elasticity of experience	0.1301	Heckman et al. (2006)[27]
η_3	Second-degree Mincer elasticity of experience	−0.0023	Heckman et al. (2006)[27]
χ_i	Fraction of treatment cost financed out of saving	Set as saving rate	World Bank (2020b)[19]

Table S2 describes some of the data lacking for 60 countries.

Table S2. Missing data for 60 countries: The columns represent region, country code, country, GDP, treatment cost, education, house expenditures, labor participation rate, physical capital, population, and saving rate, respectively

Region	Code	World Bank Country	GDP	TC	EDU	HEPC	Lab	Cap	Pop	Save
**East Asia & Pacific**	ASM	American Samoa	X			X	X	X	X	X
	PRK	Korea, Dem. People's Rep.	X		X	X		X		X
	GUM	Guam	X			X		X		X
	KIR	Kiribati					X	X		
	MHL	Marshall Islands					X	X	X	
	FSM	Micronesia, Fed. Sts.					X	X		X
	MMR	Myanmar						X		
	NRU	Nauru					X	X	X	X

	MNP	Northern Mariana Islands	X	X	X	X	X	X
	PLW	Palau			X	X	X	X
	PNG	Papua New Guinea				X		X
	WSM	Samoa				X		X
	SLB	Solomon Islands				X		
	TWN	Taiwan (Province of China)	X	X				X
	TLS	Timor-Leste				X		
	TON	Tonga				X		
	TUV	Tuvalu			X	X	X	X
	VUT	Vanuatu				X		
**Europe & Central Asia**	AND	Andorra	X	X	X	X	X	X
	ROU	Romania		X				
	GRL	Greenland	X	X	X	X	X	X
	MCO	Monaco	X	X	X	X	X	X
	SMR	San Marino			X	X	X	X
	TKM	Turkmenistan						X
**Latin America & Caribbean**	ATG	Antigua and Barbuda			X			
	CUB	Cuba	X	X		X		X
	DMA	Dominica			X		X	
	GRD	Grenada			X			X
	GUY	Guyana				X		X
	HTI	Haiti				X		
	NIC	Nicaragua				X		
	PRI	Puerto Rico		X		X		X
	KNA	St. Kitts and Nevis			X		X	X
	LCA	St. Lucia						X
	VCT	St. Vincent and the Grenadines						X
	TTO	Trinidad and Tobago						X
	VIR	Virgin Islands (U.S.)	X	X		X		X
	VEN	Venezuela, RB	X	X				
**Middle East & North Africa**	DZA	Algeria				X		
	IRN	Iran, Islamic Rep.						X
	LBY	Libya				X		X
	SYR	Syrian Arab Republic	X	X				
	ARE	United Arab Emirates				X		X
	YEM	Yemen, Rep.	X	X				X
**North America**	BMU	Bermuda	X	X	X		X	
**South Asia**	AFG	Afghanistan				X		X
	CAF	Central African Republic						X

**Sub-Saharan Africa**	TCD	Chad								X
	GNQ	Equatorial Guinea								X
	ERI	Eritrea	X			X		X		X
	LBR	Liberia								X
	MWI	Malawi								X
	STP	Sao Tome and Principe								X
	SYC	Seychelles			X		X	X		
	SOM	Somalia				X		X		X
	SSD	South Sudan	X			X		X		
**Others**	COK	Cook Islands	X		X	X	X	X	X	X
	NIU	Niue	X		X	X	X	X	X	X
	PSE	Palestine	X		X	X		X		
	TKL	Tokelau	X		X	X	X	X	X	X

D: Imputation

For the 60 countries and territories with incomplete data (mostly on education, physical capital, and the saving rate), we used a linear projection to approximate the economic burden of COPD. Thus, we used the percentage of economic loss in total GDP from 2020–2050 as the dependent variable and disability-adjusted life years (DALYs) due to COPD in 2020–2050 as the independent variable, based on results from the 144 countries with complete data. To get the adjusted DALYs, we used the age-sex fraction of each country in 2019 as a constant age-standardized rate because we lacked some age-sex-specific population data for 2020–2050. The primary ordinary linear regression model (Model 1) is as follows:

$$Y_{imputed} = \beta_0 + \beta_1 \times DALYs$$

To guarantee the model's accuracy and robustness, we also considered including country GDP per capita in 2019 (in constant 2017 INT$), country life expectancy in 2019,[29] and country population size in 2019 one by one and formulated the following three models:

Model 2:

$$\mathrm{Y}_{imputed} = \beta_0 + \beta_1 \times DALYs + \beta_2 \times GDP\_per\_capita$$

Model 3:

$$Y_{imputed} = \beta_0 + \beta_1 \times DALYs + \beta_2 \times GDP\_per\_capita + \beta_3 \times Life\_Expectancy$$

Model 4:

$$Y_{imputed} = \beta_0 + \beta_1 \times DALYs + \beta_2 \times GDP\_per\_capita + \beta_3 \times Life\_Expectancy + \beta_4 \times Population$$

Of the total 204 countries, 144 have complete data and 33 countries have data on population, GDP, and national life expectancy. These 177 countries are used for analysis in the following imputation analysis. **Table S3** presents the regression results for these four models. To prevent the impact of heteroskedasticity, we rely on robust standard errors to derive more conservative estimates of the variance.

Table S3. Estimating the percentage of economic loss with DALYs and different models

	**Model 1**		**Model 2**		**Model 3**		**Model 4**	
	**Coefficient (RSE)**	**P value**	**Coefficient (RSE)**	**P value**	**Coefficient (RSE)**	**P value**	**Coefficient (RSE)**	**P value**
**DALYs**	7.52E-07 (1.09E-07)	<0.0001	7.46E-07 (1.17E-07)	<0.0001	7.62E-07 (1.23E-07)	<0.0001	7.19E-07 (1.23E-07)	<0.0001
**GDP per capita**			3.63E-10 (1.10E-09)	0.744	1.39E-09 (1.67E-09)	0.410	1.77E-09 (1.69E-09)	0.297
**Life expectancy**					-4.40E-06 (3.70E-06)	0.237	-4.45E-06 (3.77E-06)	0.241
**Population**							3.36E-13 (4.50E-13)	0.457
**Adjusted R-square**	0.5746		0.5691		0.5796		0.6072	
**AIC**	-2,017.86		-2,016.04		-2,015.56		-2,023.36	
**BIC**	-2,008.95		-2,004.16		-2,000.72		-2,005.54	
**Total economic burden for 177 countries (in billions of 2017 INT$)**	4,301.49		4,301.53		4,301.16		4,300.41	

Note: AIC: Akaike Information Criterion. BIC: Bayes Information Criterion. RSE: Robust standard error. Of the 177 countries, 144 have complete data and 33 countries have data on population, GDP, and national life expectancy. See Table S2 for the specific countries with or without data.

The regression table shows that the Akaike Information Criterion supports Model 4, while the Bayes Information Criterion supports Model 1 as it penalizes the number of independent variables more severely. The total economic burden for these 177 countries is very similar. Therefore, considering interpretability and data completeness, we finally chose Model 1 as the imputation model.

Figure S4 presents the diagnostic plots of Model 1. Considering the distribution of residuals, we believe that the normality of the residual assumption is reasonable except for a few outliers, but the assumption of equal variance of error terms does not seem to hold. Absolute residuals are positively correlated with DALYs. Thus, we used weighted least squares to estimate the economic loss. **Table S4** shows the regression results for the weighted least squares estimation.

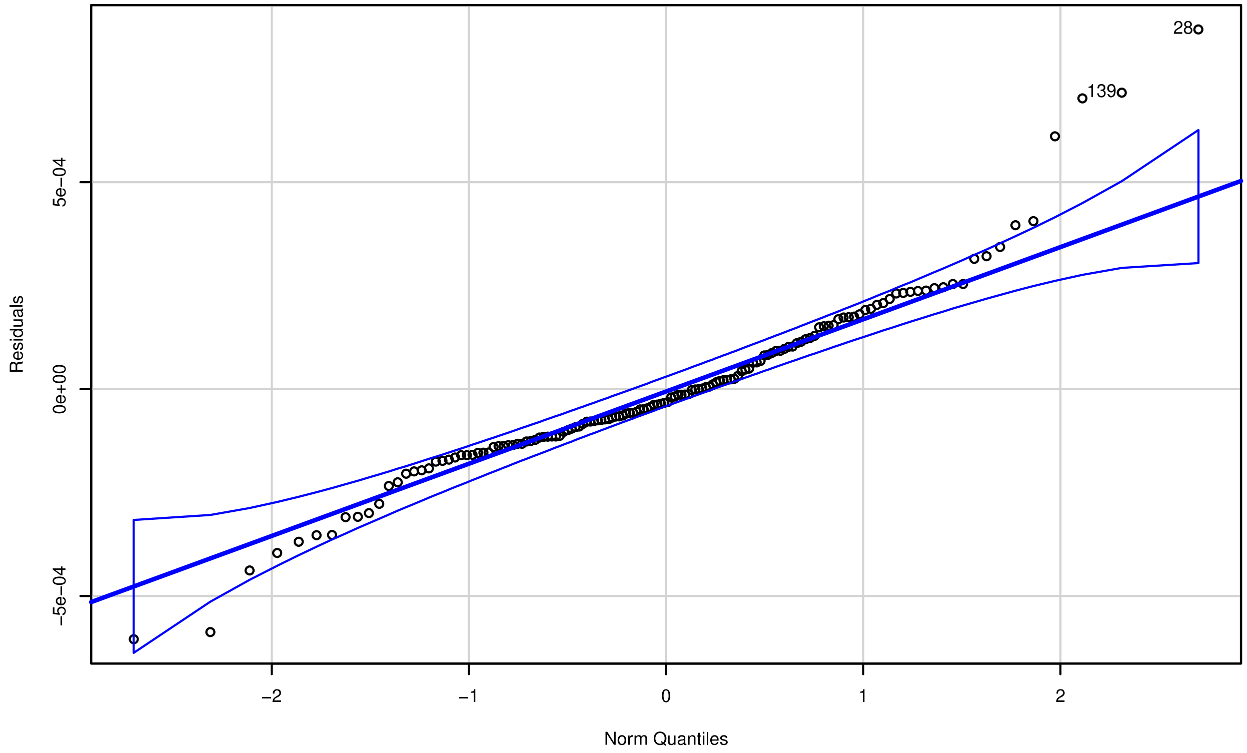

Figure S4. (a) Diagnostic plots of model: Q-Q plot of residuals

Note: Each point represents one of 144 countries; the purple line represents the regression line of the residuals on the norm quantiles; the purple area represents the point-wise confidence envelope at a 95% confidence level.

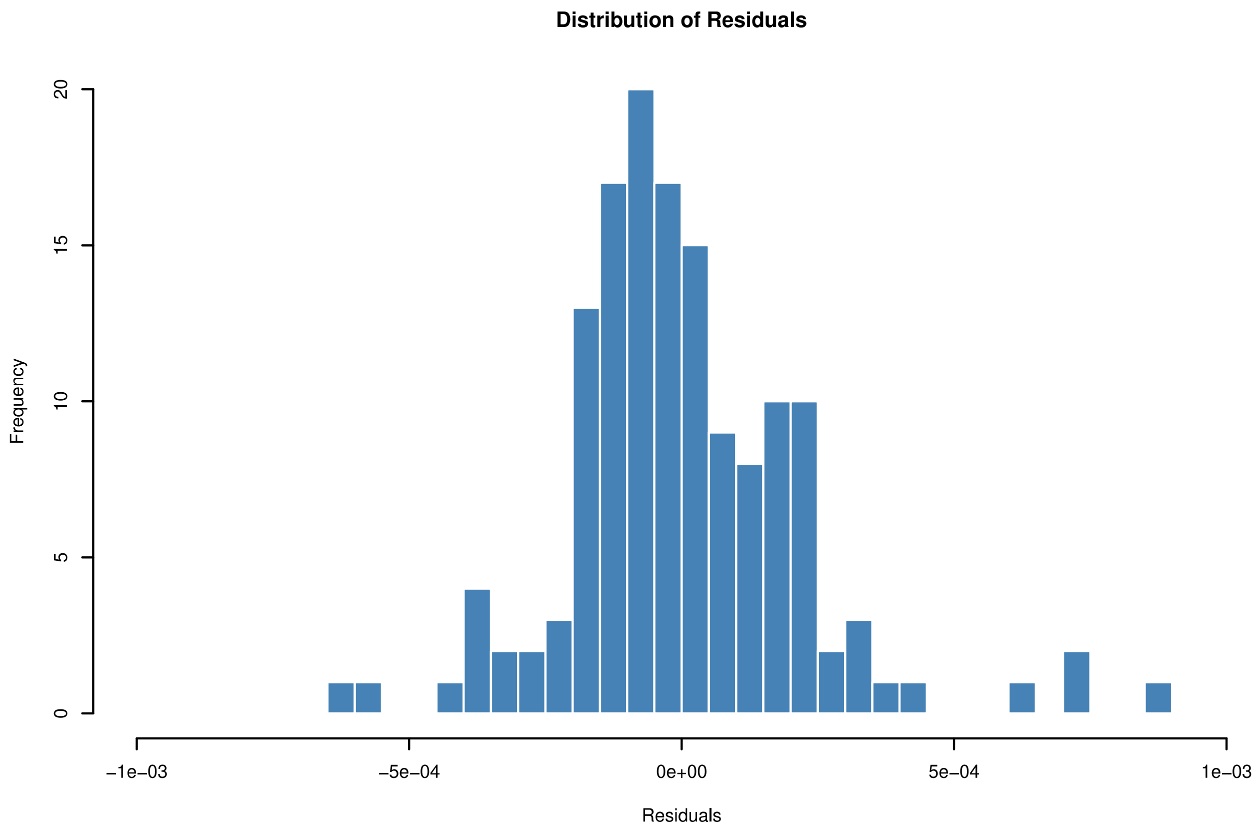

Figure S4. (b) Diagnostic plots of model: Histogram of residuals

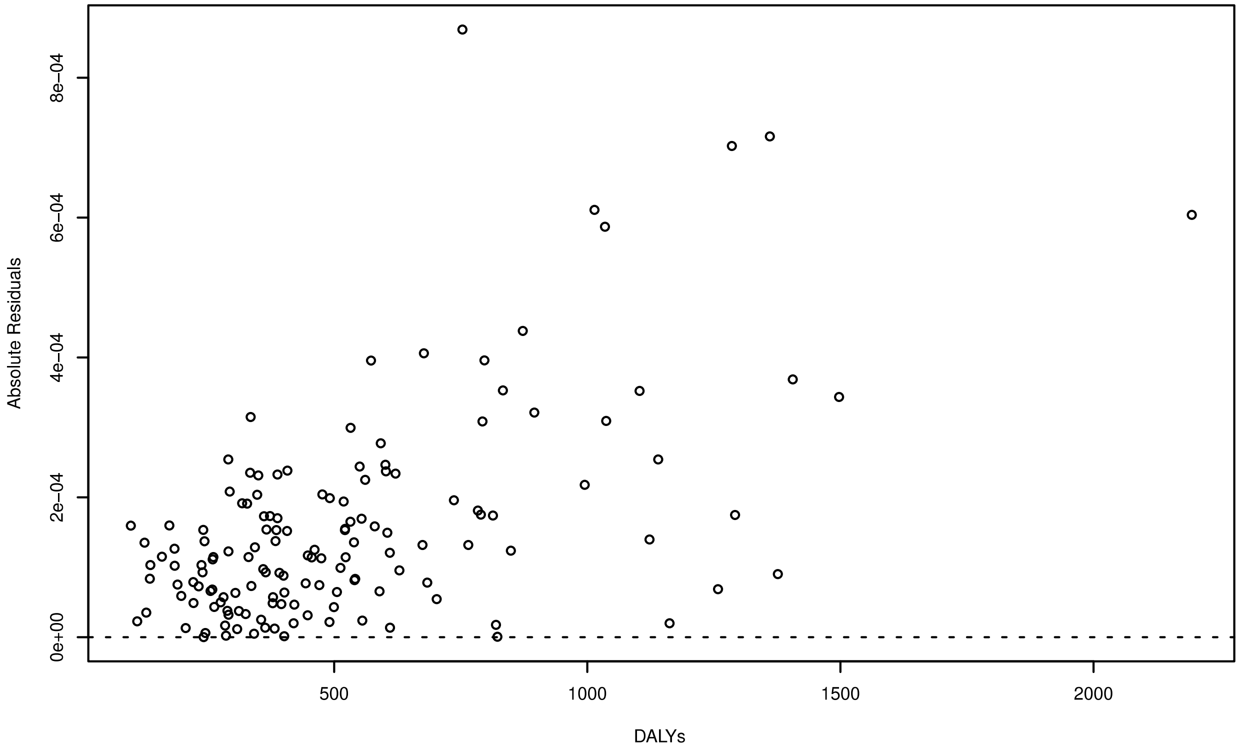

Figure S4. (c) Diagnostic plots of model: Scatter plot of absolute residuals and DALYs

Table S4. Estimating the percentage of economic loss with DALYs with weighted least squares

	**Coefficient (RSE)**	**P value**
**DALYs**	8.790E-07 (6.881E-08)	<0.0001
**AIC**	-2,087.18	
**BIC**	-2,078.27	
**Adjusted R-squared**	0.5846	
**Total economic burden for 177 countries in billions of 2017 INT$**	4,301.49	

Note: AIC: Akaike Information Criterion. BIC: Bayes Information Criterion. RSE: Robust standard error. Of the 177 countries, 144 have complete data and 33 countries have data on population, GDP, and national life expectancy. See Table S2 for the specific countries with or without data.

E: Discounted estimates

Tables S5–S6 show the total discounted economic burden of COPD in 2020–2050 for each country, by World Bank region, and by World Bank income group, using discount rates of 0%, 2%, 4%, and 5%.

Table S5. Total macroeconomic burden attributable to COPD in 2020–2050, using different discount rates, for 204 countries, by country and World Bank region (in 2017 INT$)

Region	Country	Economic burden discounted at 0% in millions of 2017 INT$ (lower and upper bound†)	Economic burden discounted at 2% in millions of 2017 INT$ (lower and upper bound†)	Economic burden discounted at 4% in millions of 2017 INT$ (lower and upper bound†)	Economic burden discounted at 5% in millions of 2017 INT$ (lower and upper bound†)
East Asia & Pacific	American Samoa*	25(17-36)	17(12-24)	11(8-16)	9(6-13)
	Australia	62,003(49,085-76,353)	41,316(32,725-50,852)	27,975(22,168-34,415)	23,179(18,371-28,509)
	Brunei Darussalam	461(299-683)	314(205-464)	218(143-321)	183(120-269)
	Cambodia	5,144(3,452-7,348)	3,286(2,206-4,690)	2,121(1,426-3,026)	1,713(1,152-2,443)
	China	2,433,192(1,838,685-3,228,014)	1,647,155(1,247,345-2,178,573)	1,134,294(860,998-1,495,253)	948,099(720,568-1,247,611)
	Fiji	175(87-320)	118(59-215)	81(41-147)	68(35-122)
	Guam*	185(127-264)	123(85-175)	83(57-118)	69(47-98)
	Indonesia	181,606(128,131-246,884)	118,454(83,727-160,655)	78,307(55,464-105,926)	64,055(45,420-86,523)
	Japan	136,608(108,052-172,309)	93,058(73,676-117,203)	64,554(51,152-81,185)	54,177(42,945-68,086)
	Kiribati*	9(6-14)	6(4-9)	4(3-6)	3(2-5)
	Korea, Dem. People's Rep.*	4,318(3,484-5,363)	2,851(2,314-3,531)	1,914(1,563-2,364)	1,579(1,294-1,948)
	Korea, Rep.	84,192(68,457-103,447)	55,846(45,392-68,593)	37,607(30,551-46,175)	31,064(25,228-38,136)
	Lao PDR	3,609(2,306-5,459)	2,329(1,487-3,521)	1,522(971-2,300)	1,237(789-1,869)
	Malaysia	34,870(21,927-52,627)	22,628(14,294-34,013)	14,873(9,445-22,255)	12,129(7,724-18,105)
	Marshall Islands*	9(5-14)	6(4-9)	4(2-6)	3(2-5)
	Micronesia, Fed. Sts.*	16(8-24)	11(6-16)	7(4-11)	6(3-9)
	Mongolia	686(421-1,101)	453(278-726)	303(187-486)	250(154-400)
	Myanmar*	19,377(15,280-23,387)	12,302(9,745-14,828)	7,935(6,313-9,551)	6,420(5,120-7,722)
	Nauru*	4(2-5)	2(2-4)	2(1-3)	1(1-2)
	New Zealand	14,207(11,119-17,849)	9,486(7,431-11,894)	6,440(5,050-8,057)	5,344(4,192-6,678)
	Northern Mariana Islands*	43(31-62)	29(21-41)	20(14-28)	16(12-23)
	Palau*	14(10-20)	9(7-13)	7(5-9)	5(4-8)
	Papua New Guinea*	2,475(1,667-3,642)	1,618(1,095-2,373)	1,075(731-1,572)	882(602-1,289)
	Philippines	59,665(44,301-81,544)	38,171(28,396-51,978)	24,684(18,403-33,475)	19,950(14,892-26,995)
	Samoa*	44(30-62)	29(20-41)	20(13-28)	16(11-23)
	Singapore	8,517(6,626-11,111)	5,664(4,407-7,382)	3,826(2,978-4,981)	3,166(2,464-4,119)
	Solomon Islands*	85(57-123)	56(38-81)	38(25-54)	31(21-45)
	Taiwan (Province of China)*	37,186(25,009-55,487)	24,746(16,666-36,854)	16,738(11,288-24,879)	13,862(9,354-20,584)
	Thailand	27,837(20,726-38,064)	18,521(13,764-25,327)	12,519(9,284-17,120)	10,364(7,677-14,173)
	Timor-Leste*	182(127-249)	124(87-170)	86(61-118)	72(51-99)
	Tonga*	19(13-27)	13(9-18)	9(6-12)	7(5-10)
	Tuvalu*	4(3-6)	3(2-4)	2(1-2)	1(1-2)
	Vanuatu*	45(31-62)	29(20-41)	20(14-27)	16(11-22)

	Vietnam	90,830(51,774-139,206)	58,123(33,033-88,791)	37,599(21,294-57,227)	30,396(17,181-46,170)
**Europe & Central Asia**	Albania	1,032(621-1,576)	674(407-1,027)	447(271-679)	366(222-555)
	Andorra*	161(118-212)	107(79-140)	72(53-94)	59(44-78)
	Armenia	1,374(1,027-1,870)	903(676-1,227)	603(451-817)	496(371-671)
	Austria	13,475(11,536-15,945)	9,034(7,712-10,707)	6,157(5,238-7,310)	5,119(4,347-6,084)
	Azerbaijan	2,424(1,579-3,587)	1,683(1,097-2,489)	1,190(777-1,760)	1,009(659-1,491)
	Belarus	1,985(1,247-3,395)	1,401(885-2,375)	1,008(640-1,694)	862(549-1,442)
	Belgium	22,505(18,246-27,405)	15,151(12,273-18,442)	10,374(8,394-12,622)	8,646(6,991-10,518)
	Bosnia and Herzegovina	1,504(1,079-2,090)	999(717-1,386)	674(484-934)	558(400-772)
	Bulgaria	13,864(9,357-19,987)	9,009(6,117-12,925)	5,930(4,053-8,461)	4,839(3,319-6,884)
	Croatia	2,818(2,042-3,921)	1,889(1,367-2,623)	1,287(931-1,784)	1,070(774-1,482)
	Cyprus	1,032(837-1,263)	658(532-808)	424(341-523)	343(275-423)
	Czech Republic	27,832(19,954-36,682)	18,006(12,944-23,707)	11,796(8,504-15,512)	9,603(6,934-12,620)
	Denmark	17,973(14,371-22,219)	12,126(9,684-14,978)	8,326(6,639-10,274)	6,950(5,537-8,573)
	Estonia	1,868(1,298-2,635)	1,214(845-1,709)	800(558-1,123)	653(456-916)
	Finland	5,573(4,487-6,900)	3,748(3,012-4,641)	2,563(2,056-3,175)	2,135(1,711-2,645)
	France	61,976(50,476-76,577)	41,073(33,366-50,877)	27,642(22,387-34,338)	22,828(18,458-28,404)
	Georgia	1,063(806-1,438)	700(530-947)	468(354-634)	385(291-522)
	Germany	358,903(271,060-446,417)	237,145(179,915-294,570)	159,117(121,304-197,354)	131,218(100,286-162,623)
	Greece	4,692(3,799-5,771)	3,186(2,566-3,930)	2,202(1,763-2,725)	1,845(1,473-2,286)
	Greenland*	145(106-195)	96(70-129)	65(48-87)	53(39-71)
	Hungary	30,972(20,225-43,961)	20,316(13,332-28,699)	13,516(8,918-18,992)	11,094(7,342-15,544)
	Iceland	753(552-977)	499(366-647)	337(247-436)	278(205-360)
	Ireland	77,650(64,612-93,941)	48,112(40,009-58,221)	29,987(24,917-36,299)	23,748(19,724-28,754)
	Italy	46,401(39,329-54,551)	31,043(26,291-36,553)	21,110(17,861-24,903)	17,531(14,824-20,701)
	Kazakhstan	22,953(16,219-31,404)	15,352(10,884-20,958)	10,433(7,422-14,207)	8,659(6,172-11,777)
	Kyrgyz Republic	1,205(828-1,836)	799(551-1,211)	538(372-811)	444(308-668)
	Latvia	1,496(945-2,341)	981(622-1,530)	653(415-1,014)	536(342-831)
	Lithuania	3,247(2,399-4,514)	2,140(1,582-2,970)	1,431(1,059-1,982)	1,178(872-1,630)
	Luxembourg	1,363(1,078-1,727)	917(725-1,161)	628(496-794)	523(414-661)
	Moldova	835(591-1,245)	560(397-832)	383(272-565)	318(226-469)
	Monaco*	375(293-486)	249(195-322)	168(132-216)	139(109-179)
	Montenegro	284(189-407)	184(123-263)	121(81-173)	98(66-140)
	Netherlands	59,151(47,282-72,511)	39,607(31,650-48,497)	26,961(21,532-32,975)	22,402(17,885-27,384)
	North Macedonia	802(515-1,208)	539(348-807)	368(239-549)	306(200-455)
	Norway	15,236(13,095-17,015)	10,385(8,908-11,607)	7,204(6,165-8,059)	6,044(5,165-6,765)
	Poland	50,423(38,801-67,560)	32,982(25,413-44,045)	21,869(16,876-29,097)	17,917(13,837-23,790)
	Portugal	6,571(5,303-8,338)	4,397(3,534-5,597)	2,992(2,393-3,822)	2,486(1,984-3,182)
	Romania*	31,017(22,163-42,379)	20,101(14,414-27,415)	13,234(9,522-18,017)	10,814(7,794-14,709)
	Russian Federation	58,148(42,128-80,218)	40,315(29,316-55,363)	28,491(20,800-38,934)	24,140(17,660-32,904)
	San Marino*	65(46-90)	44(31-61)	30(22-42)	25(18-35)

	Serbia	7,397(5,099-10,622)	4,881(3,370-6,982)	3,268(2,261-4,656)	2,692(1,864-3,827)
	Slovak Republic	7,049(4,832-10,092)	4,574(3,141-6,535)	3,006(2,069-4,285)	2,451(1,688-3,490)
	Slovenia	1,592(1,205-2,137)	1,058(799-1,422)	714(538-961)	591(444-796)
	Spain	57,633(47,876-69,965)	37,982(31,462-46,200)	25,395(20,967-30,960)	20,897(17,222-25,509)
	Sweden	21,780(19,007-24,778)	14,541(12,665-16,561)	9,862(8,570-11,246)	8,176(7,096-9,331)
	Switzerland	23,797(18,586-30,311)	16,099(12,589-20,453)	11,083(8,677-14,042)	9,264(7,257-11,721)
	Tajikistan	2,094(1,401-3,064)	1,325(885-1,940)	847(565-1,240)	680(453-996)
	Turkey	90,071(68,237-120,708)	59,263(44,872-79,264)	39,563(29,934-52,799)	32,533(24,605-43,368)
	Turkmenistan*	1,268(762-2,221)	878(526-1,532)	617(369-1,073)	521(311-904)
	Ukraine	957(583-1,505)	735(455-1,146)	585(366-903)	528(333-812)
	United Kingdom	140,317(117,693-161,846)	94,109(78,862-108,566)	64,207(53,744-74,087)	53,425(44,691-61,654)
	Uzbekistan	9,197(5,449-14,682)	5,991(3,576-9,508)	3,955(2,379-6,235)	3,232(1,953-5,078)
**Latin America & Caribbean**	Antigua and Barbuda*	30(20-44)	20(13-29)	13(9-19)	11(7-16)
	Argentina	23,775(18,963-29,751)	16,257(12,967-20,319)	11,313(9,023-14,123)	9,506(7,580-11,860)
	Bahamas, The	146(90-223)	99(61-151)	68(42-104)	57(35-86)
	Barbados	44(26-67)	30(18-45)	21(12-31)	17(10-26)
	Belize	68(44-97)	45(30-65)	31(20-44)	26(17-37)
	Bolivia	2,427(1,506-3,620)	1,610(1,000-2,400)	1,086(675-1,616)	898(559-1,336)
	Brazil	43,981(37,402-52,837)	30,462(25,906-36,560)	21,492(18,277-25,767)	18,189(15,467-21,796)
	Chile	10,949(8,803-13,757)	7,260(5,826-9,123)	4,888(3,913-6,144)	4,037(3,228-5,075)
	Colombia	19,652(12,837-30,453)	13,141(8,606-20,222)	8,932(5,866-13,641)	7,416(4,877-11,281)
	Costa Rica	2,368(1,549-3,553)	1,561(1,025-2,330)	1,044(688-1,550)	860(568-1,272)
	Cuba*	7,850(4,891-10,916)	5,198(3,254-7,211)	3,499(2,200-4,844)	2,892(1,822-3,998)
	Dominica*	16(11-24)	11(7-17)	8(5-12)	7(4-10)
	Dominican Republic	9,253(4,617-16,250)	5,892(2,958-10,306)	3,790(1,916-6,599)	3,055(1,551-5,305)
	Ecuador	1,856(1,315-2,577)	1,254(888-1,740)	862(609-1,195)	719(508-997)
	El Salvador	826(459-1,331)	551(309-883)	374(211-595)	310(176-492)
	Grenada*	39(28-53)	26(18-35)	17(12-23)	14(10-19)
	Guatemala	2,006(1,311-2,905)	1,308(857-1,891)	864(568-1,248)	707(465-1,019)
	Guyana*	1,000(618-1,509)	654(404-986)	433(267-652)	354(218-533)
	Haiti*	632(363-1,022)	433(249-698)	302(174-485)	253(146-407)
	Honduras	1,799(963-3,020)	1,182(633-1,982)	789(423-1,320)	649(348-1,084)
	Jamaica	463(267-755)	313(182-507)	216(127-347)	181(107-289)
	Mexico	38,756(28,360-52,323)	25,802(18,940-34,714)	17,453(12,854-23,393)	14,453(10,664-19,334)
	Nicaragua*	669(411-980)	457(280-669)	317(194-464)	266(163-389)
	Panama	2,816(1,996-3,932)	1,817(1,290-2,531)	1,186(844-1,648)	963(686-1,337)
	Paraguay	2,285(1,401-3,345)	1,498(924-2,188)	995(618-1,451)	817(508-1,190)
	Peru	4,133(2,990-5,777)	2,709(1,957-3,786)	1,800(1,298-2,516)	1,476(1,063-2,064)
	Puerto Rico*	2,330(1,516-3,568)	1,655(1,080-2,529)	1,194(781-1,820)	1,021(669-1,553)
	St. Kitts and Nevis*	28(19-40)	19(13-27)	13(9-18)	11(7-15)
	St. Lucia*	72(50-96)	48(34-64)	33(23-44)	28(20-37)
	St. Vincent and the Grenadines*	28(20-38)	19(13-26)	13(9-18)	11(8-15)
	Suriname	93(58-142)	64(40-98)	45(28-68)	38(24-58)

	Trinidad and Tobago*	421(235-704)	300(167-500)	217(121-360)	185(103-308)
	Uruguay	2,143(1,660-2,656)	1,461(1,132-1,810)	1,014(785-1,255)	851(659-1,053)
	Venezuela, RB*	8,671(4,881-14,038)	5,771(3,256-9,315)	3,904(2,207-6,283)	3,233(1,829-5,196)
	Virgin Islands (U.S.)*	115(74-166)	76(49-110)	52(33-75)	43(28-62)
**Middle East & North Africa**	Algeria*	9,505(6,841-13,076)	6,446(4,634-8,870)	4,444(3,192-6,116)	3,716(2,667-5,114)
	Bahrain	647(494-857)	422(322-561)	280(213-372)	229(174-305)
	Djibouti	223(139-343)	143(89-221)	93(58-144)	76(47-117)
	Egypt, Arab Rep.	61,572(37,227-90,564)	39,738(24,087-58,320)	25,947(15,770-37,986)	21,079(12,830-30,818)
	Iran, Islamic Rep.*	25,479(20,883-31,886)	17,387(14,248-21,787)	12,061(9,882-15,128)	10,113(8,285-12,690)
	Iraq	3,628(2,648-4,977)	2,354(1,718-3,228)	1,547(1,128-2,120)	1,261(920-1,728)
	Israel	15,654(11,952-20,387)	10,335(7,905-13,437)	6,925(5,307-8,987)	5,706(4,377-7,397)
	Jordan	1,009(692-1,443)	682(469-972)	469(323-665)	391(270-555)
	Kuwait	1,721(1,348-2,200)	1,153(903-1,472)	784(614-1,000)	650(510-829)
	Lebanon	194(123-284)	144(92-211)	110(71-161)	97(63-142)
	Libya*	9,878(6,502-14,080)	5,769(3,801-8,214)	3,374(2,226-4,799)	2,587(1,707-3,677)
	Malta	1,333(1,102-1,611)	845(699-1,020)	540(447-652)	434(359-524)
	Morocco	6,211(4,373-8,807)	4,102(2,882-5,824)	2,749(1,927-3,909)	2,265(1,586-3,224)
	Oman	1,999(1,307-2,868)	1,311(855-1,884)	872(567-1,256)	715(464-1,032)
	Qatar	1,328(988-1,794)	884(658-1,194)	597(445-806)	494(369-666)
	Saudi Arabia	22,810(16,904-31,512)	15,217(11,255-21,018)	10,306(7,607-14,231)	8,537(6,294-11,786)
	Syrian Arab Republic*	1,457(924-2,294)	971(616-1,525)	658(418-1,030)	545(346-853)
	Tunisia	2,206(1,633-2,979)	1,477(1,090-1,998)	1,005(739-1,362)	835(613-1,133)
	United Arab Emirates*	23,433(14,336-37,173)	15,537(9,533-24,573)	10,469(6,442-16,510)	8,654(5,332-13,627)
	Yemen, Rep.*	1,875(1,222-2,764)	1,252(815-1,843)	849(553-1,249)	704(459-1,035)
**North America**	Bermuda*	136(95-194)	91(64-129)	62(43-88)	51(36-73)
	Canada	63,972(48,724-81,122)	43,129(32,871-54,614)	29,586(22,562-37,410)	24,685(18,829-31,192)
	United States	1,844,940(1,542,971-2,093,179)	1,251,276(1,046,747-1,417,831)	864,084(722,982-977,785)	723,529(605,416-818,172)
**South Asia**	Afghanistan*	1,105(682-1,607)	758(467-1,103)	530(327-771)	447(275-649)
	Bangladesh	54,743(49,609-79,573)	35,597(31,746-52,023)	23,458(20,545-34,490)	19,158(16,616-28,259)
	Bhutan	632(386-1,050)	410(251-681)	270(165-447)	220(135-365)
	India	794,589(559,659-1,063,324)	516,142(364,578-688,896)	339,701(240,717-452,066)	277,235(196,790-368,352)
	Maldives	326(234-447)	206(147-282)	131(94-179)	105(75-143)
	Nepal	10,423(6,565-15,511)	6,831(4,305-10,144)	4,545(2,867-6,733)	3,733(2,355-5,522)
	Pakistan	61,060(42,544-85,291)	40,653(28,328-56,798)	27,507(19,169-38,442)	22,786(15,880-31,848)
	Sri Lanka	13,005(8,065-21,194)	8,687(5,400-14,063)	5,900(3,677-9,480)	4,896(3,056-7,836)
**Sub-Saharan Africa**	Angola	2,325(1,454-3,419)	1,641(1,027-2,413)	1,181(739-1,736)	1,009(632-1,484)
	Benin	1,671(1,045-2,567)	1,096(686-1,682)	729(457-1,117)	598(375-916)
	Botswana	866(622-1,196)	573(411-793)	385(275-534)	317(227-441)
	Burkina Faso	1,762(1,127-2,658)	1,138(729-1,715)	745(478-1,120)	606(389-911)
	Burundi	158(96-253)	109(67-175)	77(47-123)	65(40-104)
	Cabo Verde	91(61-130)	60(40-86)	40(27-57)	33(22-47)
	Cameroon	3,036(1,940-4,482)	1,996(1,275-2,946)	1,332(850-1,965)	1,094(699-1,615)

	Central African Republic*	152(92-243)	100(60-159)	66(40-106)	54(33-87)
	Chad*	369(241-544)	257(168-379)	182(118-268)	154(100-227)
	Comoros	68(41-107)	46(27-71)	31(19-48)	26(16-40)
	Congo, Dem. Rep.	4,336(2,524-6,862)	2,870(1,667-4,548)	1,929(1,118-3,061)	1,592(922-2,528)
	Congo, Rep.	184(110-274)	137(81-204)	104(62-155)	91(54-136)
	Côte d'Ivoire	5,512(3,556-8,322)	3,522(2,271-5,315)	2,276(1,466-3,432)	1,839(1,184-2,772)
	Equatorial Guinea*	145(88-224)	112(68-172)	87(53-134)	77(47-118)
	Eritrea*	230(146-338)	154(97-226)	104(66-153)	87(55-127)
	Eswatini	227(155-323)	155(105-221)	108(73-154)	91(61-130)
	Ethiopia	11,882(7,920-16,605)	7,554(5,039-10,556)	4,851(3,239-6,779)	3,907(2,610-5,459)
	Gabon	401(275-568)	268(183-379)	182(124-257)	151(103-213)
	Gambia, The	215(129-340)	140(84-221)	92(56-145)	75(46-119)
	Ghana	8,961(5,365-13,627)	5,881(3,518-8,933)	3,914(2,340-5,939)	3,213(1,920-4,873)
	Guinea	2,076(1,262-3,229)	1,323(805-2,056)	852(519-1,323)	687(419-1,067)
	Guinea-Bissau	174(105-274)	114(69-179)	76(46-119)	62(38-98)
	Kenya	6,287(4,605-8,403)	4,102(3,001-5,488)	2,712(1,982-3,634)	2,219(1,621-2,975)
	Lesotho	171(106-270)	119(74-188)	84(52-133)	72(44-113)
	Liberia*	101(66-152)	71(45-105)	50(32-74)	42(27-63)
	Madagascar	1,576(923-2,508)	1,044(612-1,661)	702(412-1,117)	580(340-922)
	Malawi*	650(434-946)	429(286-625)	288(191-419)	237(157-346)
	Mali	1,866(1,056-3,072)	1,220(692-2,005)	808(460-1,327)	662(377-1,086)
	Mauritania	555(373-806)	362(243-526)	239(161-348)	196(132-284)
	Mauritius	868(659-1,142)	564(428-742)	372(282-489)	303(230-399)
	Mozambique	1,152(799-1,698)	748(518-1,102)	492(340-724)	401(277-590)
	Namibia	384(253-564)	264(174-388)	185(122-272)	156(103-230)
	Niger	1,310(801-2,043)	839(513-1,309)	543(332-848)	439(269-686)
	Nigeria	9,359(6,399-12,788)	6,601(4,517-9,017)	4,748(3,252-6,485)	4,059(2,781-5,543)
	Rwanda	1,872(1,161-2,990)	1,202(746-1,917)	781(485-1,243)	633(393-1,006)
	Sao Tome and Principe*	38(25-55)	25(17-37)	17(11-24)	14(9-20)
	Senegal	2,628(1,750-3,798)	1,674(1,115-2,419)	1,077(718-1,557)	868(578-1,255)
	Seychelles*	102(76-138)	67(49-90)	44(33-59)	36(27-49)
	Sierra Leone	186(116-290)	129(80-200)	91(56-141)	77(48-119)
	Somalia*	473(278-796)	313(184-524)	210(123-351)	173(102-289)
	South Africa	14,352(11,523-17,978)	9,861(7,905-12,354)	6,901(5,524-8,648)	5,817(4,652-7,291)
	South Sudan*	409(264-611)	274(177-409)	187(120-278)	155(100-231)
	Sudan	3,310(2,089-4,997)	2,263(1,422-3,422)	1,574(985-2,385)	1,322(826-2,006)
	Tanzania	7,152(4,519-10,723)	4,597(2,908-6,886)	2,990(1,894-4,473)	2,424(1,537-3,624)
	Togo	1,093(659-1,683)	718(433-1,106)	479(289-737)	394(238-606)
	Uganda	3,973(2,387-6,288)	2,583(1,554-4,082)	1,702(1,025-2,685)	1,389(838-2,190)
	Zambia	1,730(1,115-2,574)	1,157(745-1,720)	786(506-1,168)	653(420-969)
	Zimbabwe	825(532-1,197)	563(363-816)	391(252-567)	328(212-476)
**Others**	Cook Islands*	15(11-19)	10(8-13)	7(5-9)	5(4-7)
	Niue*	1(0-1)	0(0-1)	0(0-0)	0(0-0)
	Palestine*	437(288-647)	294(192-435)	201(131-297)	167(109-247)
	Tokelau*	0(0-0)	0(0-0)	0(0-0)	0(0-0)

*Please note that results for countries marked with an asterisk are imputed due to missing data.

†Uncertainty intervals in parentheses are calculated based on the lower and upper bounds of 95% uncertainty intervals for GBD mortality and morbidity data.

Table S6. Total macroeconomic burden attributable to COPD in 2020–2050, using different discount rates, by World Bank region, by World Bank income group, and globally (in 2017 INT$)

	Economic burden discounted at 0% in billions of 2017 INT$ (lower and upper bound†)	Economic burden discounted at 2% in billions of 2017 INT$ (lower and upper bound†)	Economic burden discounted at 4% in billions of 2017 INT$ (lower and upper bound†)	Economic burden discounted at 5% in billions of 2017 INT$ (lower and upper bound†)
**By World Bank region**				
**East Asia & Pacific**	3,208(2,401-4,271)	2,157(1,619-2,863)	1,475(1,110-1,951)	1,228(925-1,622)
**Europe & Central Asia**	1,314(1,021-1,659)	870(677-1,097)	585(455-736)	483(376-607)
**Latin America & Caribbean**	192(140-263)	129(94-176)	88(65-120)	74(54-100)
**Middle East & North Africa**	192(132-272)	126(87-178)	84(58-118)	69(48-97)
**North America**	1,909(1,592-2,174)	1,294(1,080-1,473)	894(746-1,015)	748(624-849)
**South Asia**	936(668-1,268)	609(435-824)	402(288-543)	329(235-443)
**Sub-Saharan Africa**	107(71-155)	71(47-103)	48(32-69)	39(26-57)
**By World Bank country income group**				
**Low income**	48(31-71)	31(20-47)	21(13-31)	17(11-25)
**Lower-middle income**	1,474(1,031-2,028)	959(672-1,317)	633(444-867)	517(363-707)
**Upper-middle income**	2,925(2,195-3,904)	1,975(1,486-2,627)	1,356(1,023-1,798)	1,132(855-1,498)
**High income**	3,403(2,763-4,045)	2,285(1,857-2,713)	1,561(1,270-1,850)	1,300(1,058-1,539)
**Global (204 countries and territories)**	7,858(6,025-10,063)	5,257(4,039-6,713)	3,576(2,753-4,553)	2,970(2,289-3,776)

†Uncertainty intervals in parentheses are calculated based on the lower and upper bounds of 95% uncertainty intervals for GBD mortality and morbidity data

F: Sensitivity analysis on parameters

Tables S7–S8 show the total discounted economic burden of COPD in 2020–2050 for 204 countries, by country, by World Bank region, and by World Bank income group via probabilistic sensitivity analysis on parameters in **Table S1**. For each country, we drew 200 random samples from the uniform distributions of each parameter within a range of 50% to 150% of the initial value and estimated the economic burden of COPD. We used the same random scaling for the three parameters in the Mincer equation because we want to maintain the relative size relationship among these three parameters. Results were aggregated to compute regional and country mean values with a 95% uncertainty interval (UI). All future year estimates were discounted using an annual discount rate of 3%.

Table S7. Total macroeconomic burden attributable to COPD in 2020–2050, using 50% to 150% of parameters, for 204 countries, by country and World Bank region (in 2017 INT$)

Region	Country	Economic cost in millions of 2017 INT$ (95% UI†)	Percentage of total GDP in 2020–2050 (x 10^{-3}) (95% UI†)	Per capita loss in 2017 INT$ (95% UI†)
**East Asia & Pacific**	American Samoa*	14(9-20)	68(46-97)	250(170-360)
	Australia	34,851(25,940-44,720)	100(74-128)	1,189(885-1,525)
	Brunei Darussalam	270(198-399)	41(30-61)	567(417-840)
	Cambodia	2,699(2,048-3,726)	81(61-111)	138(105-191)
	China	1,418,649(1,026,750-2,199,858)	170(123-264)	980(710-1,520)
	Fiji	96(59-137)	35(21-49)	97(59-138)
	Guam*	102(71-144)	58(40-81)	555(384-782)
	Indonesia	96,628(51,413-168,465)	79(42-137)	315(167-549)

	Japan	81,656(52,732-121,916)	73(47-109)	699(451-1,044)
	Kiribati*	5(3-7)	67(43-100)	33(21-50)
	Korea, Dem. People's Rep.*	2,121(1,586-2,760)	173(130-226)	80(60-104)
	Korea, Rep.	47,562(28,826-72,791)	76(46-116)	950(576-1,454)
	Lao PDR	1,973(1,433-2,603)	85(62-112)	231(168-305)
	Malaysia	18,877(13,379-26,311)	61(43-85)	508(360-708)
	Marshall Islands*	5(3-7)	75(45-115)	70(42-108)
	Micronesia, Fed. Sts.*	8(5-13)	92(50-146)	65(35-102)
	Mongolia	365(221-466)	27(16-34)	94(57-120)
	Myanmar*	9,264(6,899-11,570)	105(78-132)	156(116-195)
	Nauru*	2(1-3)	57(38-83)	190(127-278)
	New Zealand	7,964(5,287-11,965)	122(81-184)	1,509(1,002-2,267)
	Northern Mariana Islands*	24(17-34)	62(44-89)	385(272-551)
	Palau*	7(5-11)	118(80-172)	399(272-580)
	Papua New Guinea*	1,234(801-1,863)	108(70-163)	106(69-161)
	Philippines	29,730(14,495-44,507)	76(37-114)	231(112-345)
	Samoa*	24(16-33)	70(47-98)	102(68-143)
	Singapore	4,999(3,411-7,670)	31(21-48)	795(542-1,219)
	Solomon Islands*	43(28-64)	94(61-138)	45(29-66)
	Taiwan (Province of China)*	20,438(13,933-30,120)	58(40-86)	866(590-1,276)
	Thailand	15,133(10,228-23,613)	43(29-67)	219(148-341)
	Timor-Leste*	99(68-138)	84(57-117)	59(40-82)
	Tonga*	11(7-15)	61(42-86)	89(61-124)
	Tuvalu*	2(1-3)	91(60-133)	144(94-209)
	Vanuatu*	23(15-32)	90(61-128)	53(36-76)
	Vietnam	47,972(36,423-63,147)	115(87-151)	456(346-600)
**Europe & Central Asia**	Albania	586(345-1,050)	51(30-92)	217(128-390)
	Andorra*	84(60-113)	82(59-111)	1,082(769-1,455)
	Armenia	736(614-901)	58(48-71)	252(210-308)
	Austria	7,399(5,460-9,771)	64(47-85)	808(596-1,067)
	Azerbaijan	1,419(1,295-1,545)	43(39-47)	131(120-143)
	Belarus	1,179(1,010-1,301)	32(28-36)	130(111-143)
	Belgium	12,622(9,849-16,079)	92(71-117)	1,053(822-1,342)
	Bosnia and Herzegovina	833(626-1,064)	59(44-75)	277(208-353)
	Bulgaria	7,313(5,279-9,647)	158(114-208)	1,189(858-1,568)
	Croatia	1,552(1,313-1,814)	48(40-56)	414(350-484)
	Cyprus	531(417-684)	44(34-56)	410(322-529)
	Czech Republic	14,412(10,502-18,578)	116(84-149)	1,351(984-1,741)
	Denmark	10,225(7,664-13,130)	117(88-150)	1,688(1,265-2,167)
	Estonia	1,000(726-1,363)	67(49-91)	801(582-1,093)
	Finland	3,143(2,275-4,310)	49(35-67)	566(410-776)
	France	34,310(24,418-45,873)	50(35-67)	513(365-685)
	Georgia	582(427-780)	31(23-42)	154(113-207)
	Germany	195,967(147,002-251,527)	189(142-243)	2,378(1,784-3,052)
	Greece	2,671(1,816-3,801)	40(27-57)	275(187-391)
	Greenland*	74(52-102)	101(70-139)	1,326(923-1,830)
	Hungary	16,669(13,383-20,742)	169(135-210)	1,831(1,470-2,278)
	Iceland	425(318-561)	72(54-95)	1,168(873-1,543)
	Ireland	39,474(24,949-53,515)	125(79-169)	7,372(4,659-9,995)
	Italy	26,427(18,469-39,064)	51(35-75)	456(319-674)
	Kazakhstan	12,026(6,570-16,425)	85(46-116)	560(306-764)
	Kyrgyz Republic	656(519-806)	68(54-83)	83(66-103)
	Latvia	821(551-1,200)	49(33-72)	494(332-723)
	Lithuania	1,749(1,246-2,554)	55(39-80)	731(520-1,067)
	Luxembourg	753(619-936)	37(30-46)	1,053(865-1,309)
	Moldova	466(378-596)	45(36-57)	124(101-159)
	Monaco*	196(146-259)	83(62-110)	4,573(3,418-6,044)
	Montenegro	154(103-230)	45(30-67)	251(168-374)
	Netherlands	32,630(24,049-41,882)	131(97-169)	1,879(1,385-2,411)
	North Macedonia	443(378-519)	47(40-55)	222(189-260)
	Norway	8,535(6,240-11,572)	98(71-133)	1,410(1,031-1,911)
	Poland	26,662(22,037-32,353)	64(53-78)	741(613-900)

	Portugal	3,656(2,661-4,836)	42(31-56)	377(274-498)
	Romania*	15,663(10,875-21,755)	82(57-114)	881(611-1,223)
	Russian Federation	33,859(27,772-40,566)	42(35-51)	240(197-287)
	San Marino*	35(25-50)	72(51-101)	1,034(728-1,456)
	Serbia	4,135(2,843-5,599)	101(69-137)	520(358-704)
	Slovak Republic	3,750(2,902-4,769)	76(59-96)	710(549-902)
	Slovenia	877(707-1,114)	36(29-46)	433(349-550)
	Spain	31,630(23,965-42,033)	69(53-92)	694(526-922)
	Sweden	11,832(8,271-17,184)	81(57-118)	1,096(766-1,591)
	Switzerland	13,173(10,443-16,910)	88(70-113)	1,412(1,119-1,812)
	Tajikistan	1,012(475-1,402)	60(28-84)	79(37-110)
	Turkey	48,030(32,409-69,533)	56(38-81)	525(354-760)
	Turkmenistan*	815(550-1,314)	37(25-60)	115(78-186)
	Ukraine	649(441-922)	24(16-34)	16(11-23)
	United Kingdom	77,263(57,678-100,593)	108(80-140)	1,083(808-1,410)
	Uzbekistan	4,831(4,298-5,282)	44(39-48)	124(111-136)
**Latin America & Caribbean**	Antigua and Barbuda*	18(13-25)	35(25-48)	171(123-232)
	Argentina	13,327(8,636-18,343)	67(43-92)	264(171-363)
	Bahamas, The	83(64-106)	29(23-37)	191(148-242)
	Barbados	25(17-38)	30(21-45)	89(61-131)
	Belize	37(27-50)	64(46-85)	76(54-101)
	Bolivia	1,377(931-2,333)	48(33-82)	99(67-168)
	Brazil	26,065(17,698-35,745)	41(28-56)	116(79-159)
	Chile	6,189(3,904-9,202)	53(33-78)	313(197-465)
	Colombia	11,186(7,782-17,337)	55(38-86)	207(144-320)
	Costa Rica	1,331(984-1,768)	42(31-56)	240(178-319)
	Cuba*	4,031(2,467-5,737)	96(59-137)	370(226-527)
	Dominica*	10(6-14)	53(36-78)	132(88-193)
	Dominican Republic	4,975(3,734-6,831)	62(47-85)	414(311-568)
	Ecuador	1,080(697-1,735)	26(17-41)	52(34-84)
	El Salvador	476(324-717)	34(23-51)	70(48-105)
	Grenada*	22(16-29)	47(34-62)	191(140-253)
	Guatemala	1,089(648-1,839)	24(14-40)	48(29-81)
	Guyana*	572(385-822)	42(28-60)	695(468-1,000)
	Haiti*	374(233-583)	49(31-77)	28(18-44)
	Honduras	1,028(663-1,705)	62(40-103)	85(55-142)
	Jamaica	269(176-402)	45(30-68)	89(58-133)
	Mexico	21,373(11,164-37,570)	38(20-66)	148(77-260)
	Nicaragua*	406(271-571)	43(28-60)	53(35-74)
	Panama	1,506(882-2,198)	39(23-57)	292(171-426)
	Paraguay	1,240(907-1,846)	47(34-70)	151(110-224)
	Peru	2,167(974-3,542)	20(09-32)	58(26-95)
	Puerto Rico*	1,376(897-2,107)	70(46-107)	500(326-766)
	St. Kitts and Nevis*	16(11-22)	49(35-68)	287(203-398)
	St. Lucia*	39(27-53)	73(50-98)	208(144-281)
	St. Vincent and the Grenadines*	16(12-22)	45(33-60)	145(107-194)
	Suriname	54(47-59)	31(27-34)	83(73-91)
	Trinidad and Tobago*	272(170-430)	43(27-68)	195(122-308)
	Uruguay	1,225(786-1,653)	68(44-92)	342(219-461)
	Venezuela, RB*	4,772(2,856-7,577)	58(35-92)	140(84-222)
	Virgin Islands (U.S.)*	64(43-91)	54(36-77)	660(441-939)
**Middle East & North Africa**	Algeria*	5,595(4,110-7,511)	47(34-63)	106(78-142)
	Bahrain	333(162-471)	17(08-24)	160(78-227)
	Djibouti	116(96-138)	45(37-53)	100(82-119)
	Egypt, Arab Rep.	31,914(16,748-47,865)	64(34-97)	244(128-366)
	Iran, Islamic Rep.*	14,756(11,793-18,413)	54(43-67)	155(124-194)
	Iraq	1,862(973-2,702)	16(08-23)	34(18-49)
	Israel	8,525(6,175-11,804)	70(51-97)	799(579-1,107)
	Jordan	561(357-665)	21(13-24)	49(31-59)
	Kuwait	934(267-1,656)	22(06-38)	190(54-337)
	Lebanon	129(84-208)	31(20-50)	20(13-33)
	Libya*	4,483(3,028-6,304)	55(37-77)	572(387-805)
	Malta	689(469-927)	77(52-103)	1,561(1,063-2,100)
	Morocco	3,481(2,307-5,257)	45(30-69)	83(55-125)
	Oman	1,089(867-1,281)	27(21-32)	177(141-209)

	Qatar*	627(1,282-1,234)	10(21-20)	182(372-358)
	Saudi Arabia	11,851(5,683-16,661)	29(14-41)	292(140-411)
	Syrian Arab Republic*	816(539-1,252)	53(35-81)	30(20-45)
	Tunisia	1,207(857-1,472)	42(30-51)	93(66-113)
	United Arab Emirates*	12,516(7,799-19,768)	68(43-108)	1,192(742-1,882)
	Yemen, Rep.*	1,074(734-1,536)	48(33-68)	27(19-39)
**North America**	Bermuda*	78(56-108)	49(35-67)	1,325(950-1,840)
	Canada	35,421(24,923-48,371)	80(56-109)	843(593-1,151)
	United States	1,060,814(818,095-1,301,134)	199(153-244)	2,969(2,290-3,642)
**South Asia**	Afghanistan*	674(450-943)	43(29-61)	13(9-18)
	Bangladesh	29,628(22,089-40,435)	66(49-90)	163(121-222)
	Bhutan	340(222-559)	108(71-178)	396(259-653)
	India	424,522(277,264-604,814)	101(66-145)	276(180-393)
	Maldives	204(93-420)	74(34-151)	374(171-768)
	Nepal	5,968(3,853-9,445)	134(87-212)	178(115-281)
	Pakistan	35,214(25,088-51,003)	95(68-138)	125(89-181)
	Sri Lanka	7,622(5,307-10,943)	92(64-132)	347(242-498)
**Sub-Saharan Africa**	Angola	1,451(1,052-2,046)	33(24-47)	27(20-39)
	Benin	906(685-1,243)	52(39-72)	51(38-70)
	Botswana	445(85-815)	42(08-78)	150(29-275)
	Burkina Faso	918(793-1,109)	46(40-56)	29(25-35)
	Burundi	96(65-134)	44(29-61)	5(4-7)
	Cabo Verde	49(40-60)	46(38-56)	78(64-95)
	Cameroon	1,660(1,092-2,306)	47(31-65)	44(29-61)
	Central African Republic*	83(53-129)	54(35-85)	13(8-20)
	Chad*	233(164-328)	41(29-57)	9(7-13)
	Comoros	39(29-53)	49(36-66)	33(25-45)
	Congo, Dem. Rep.	2,466(1,827-3,537)	60(44-86)	18(13-25)
	Congo, Rep.	121(91-165)	39(29-53)	15(11-21)
	Côte d'Ivoire	2,763(1,608-3,902)	38(22-53)	72(42-102)
	Equatorial Guinea*	109(74-157)	38(26-55)	52(35-75)
	Eritrea*	134(90-189)	45(30-64)	28(19-40)
	Eswatini	131(102-172)	52(40-68)	93(73-122)
	Ethiopia	6,238(5,101-8,203)	36(29-47)	39(32-51)
	Gabon	204(11-383)	23(01-43)	68(4-127)
	Gambia, The	116(89-160)	55(42-76)	32(25-44)
	Ghana	4,805(4,076-5,615)	69(59-81)	116(98-136)
	Guinea	1,078(844-1,359)	58(45-73)	56(44-70)
	Guinea-Bissau	97(76-125)	70(54-90)	35(28-46)
	Kenya	3,413(1,874-5,515)	37(20-59)	47(26-76)
	Lesotho	104(69-148)	100(66-143)	43(28-61)
	Liberia*	66(47-92)	36(26-50)	9(7-13)
	Madagascar	876(673-1,252)	66(51-95)	22(17-31)
	Malawi*	385(277-532)	38(27-53)	14(10-19)
	Mali	1,005(844-1,162)	58(48-67)	32(27-37)
	Mauritania	296(173-439)	33(19-49)	44(26-65)
	Mauritius	451(296-581)	64(42-82)	361(237-465)
	Mozambique	621(401-861)	40(26-56)	13(8-18)
	Namibia	230(130-345)	46(26-69)	71(40-106)
	Niger	657(373-1,061)	44(25-71)	15(9-25)
	Nigeria	5,670(4,804-6,614)	22(18-25)	19(16-22)
	Rwanda	1,018(767-1,461)	68(51-98)	57(43-81)
	Sao Tome and Principe*	20(13-29)	72(47-105)	67(44-98)
	Senegal	1,313(619-1,899)	48(23-70)	54(25-78)
	Seychelles*	54(39-73)	64(46-86)	521(380-705)
	Sierra Leone	111(61-181)	35(19-56)	11(6-17)
	Somalia*	271(174-433)	45(29-72)	11(7-18)
	South Africa	8,227(6,193-10,295)	48(36-60)	120(91-151)
	South Sudan*	245(171-347)	40(28-57)	16(11-23)
	Sudan	1,968(1,550-2,608)	43(34-57)	32(25-42)
	Tanzania	3,781(3,031-4,812)	49(40-63)	41(33-52)
	Togo	609(515-767)	80(68-101)	52(44-66)
	Uganda	2,146(1,738-2,616)	49(39-59)	32(26-39)
	Zambia	971(778-1,258)	52(42-67)	35(28-45)

	Zimbabwe	474(310-685)	37(24-54)	25(16-36)
**Others**	Cook Islands*	8(6-10)	84(62-112)	443(329-591)
	Niue*	0(0-0)	107(74-148)	194(133-268)
	Palestine*	274(198-378)	35(25-48)	39(28-54)
	Tokelau*	0(0-0)	75(53-104)	121(85-166)

*Please note that results for countries marked with an asterisk are imputed due to missing data.

†Uncertainty intervals in parentheses are calculated based on the lower and upper bounds of 95% uncertainty intervals of the burden by varying parameters.

Table S8. Total macroeconomic burden attributable to COPD in 2020–2050, using 50% to 150% of parameters, by World Bank region, by World Bank income group, and globally (in 2017 INT$)

	Economic cost in billions of 2017 INT$ (95% UI†)	Percentage of total GDP in 2020–2050 (x 10^{-3}) (95% UI†)	Per capita loss in 2017 INT$ (95% UI†)
**By World Bank region**			
**East Asia & Pacific**	1,843(1,296-2,839)	133(93-204)	749(527-1,154)
**Europe & Central Asia**	716(527-941)	87(64-115)	769(567-1,011)
**Latin America & Caribbean**	108(68-163)	46(29-69)	151(95-227)
**Middle East & North Africa**	103(64-147)	48(30-68)	183(115-262)
**North America**	1,096(843-1,350)	189(146-233)	2,745(2,111-3,379)
**South Asia**	504(334-719)	98(65-139)	239(158-340)
**Sub-Saharan Africa**	59(44-78)	42(31-55)	36(27-48)
**By World Bank region**			
**Low income**	26(20-35)	49(37-65)	27(21-37)
**Lower-middle income**	789(512-1,146)	85(55-123)	200(130-291)
**Upper-middle income**	1,688(1,211-2,582)	123(88-188)	637(457-974)
**High income**	1,921(1,432-2,466)	125(94-161)	1,550(1,156-1,991)
**Total**	4,430(3,178-6,237)	114(81-160)	502(360-706)

†Uncertainty intervals in parentheses are calculated based on the lower and upper bounds of 95% uncertainty intervals of the burden by varying parameters.

G: Contribution of treatment costs

Figures S5–S6 show the contribution of treatment costs to the total macroeconomic burden of COPD by country income group and by World Bank region.

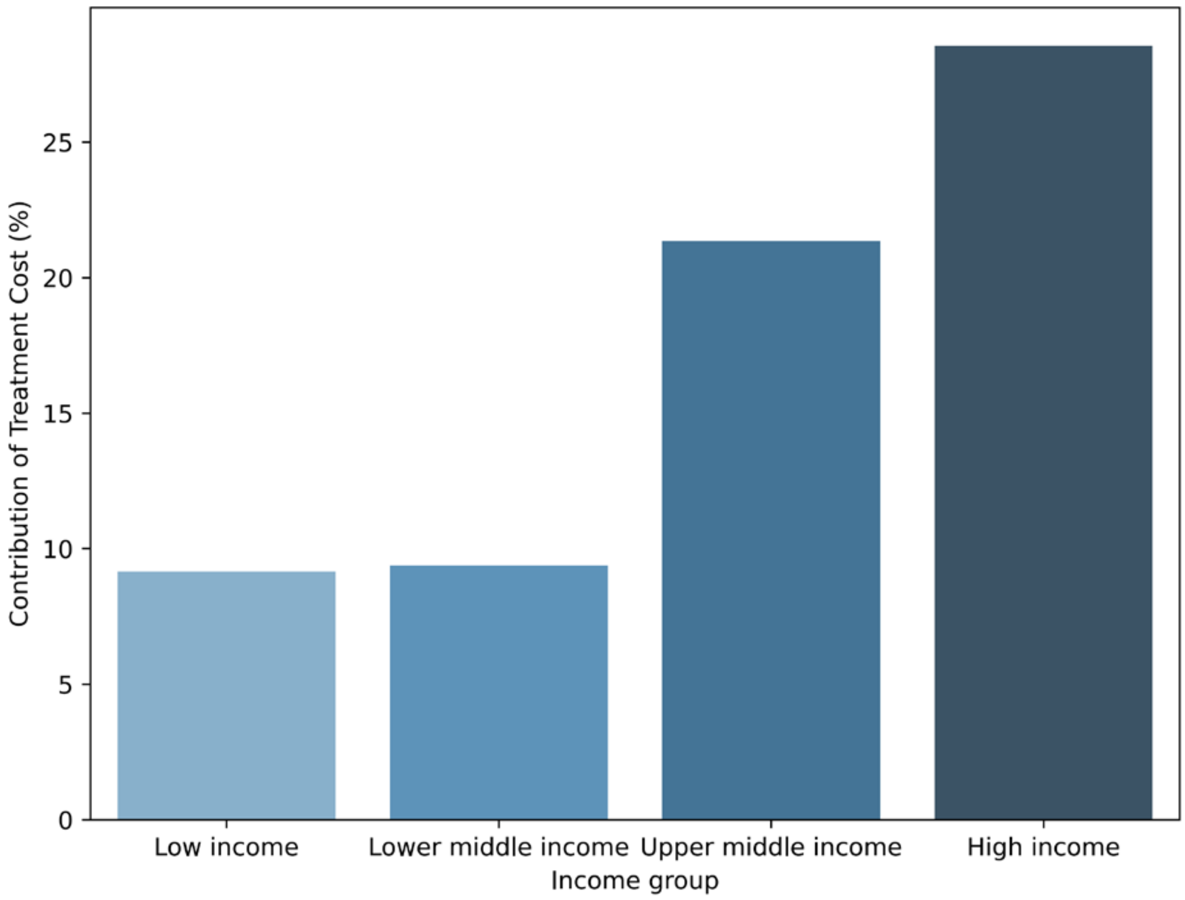

Figure S5. Contribution of treatment costs to the total economic loss due to COPD by country income group

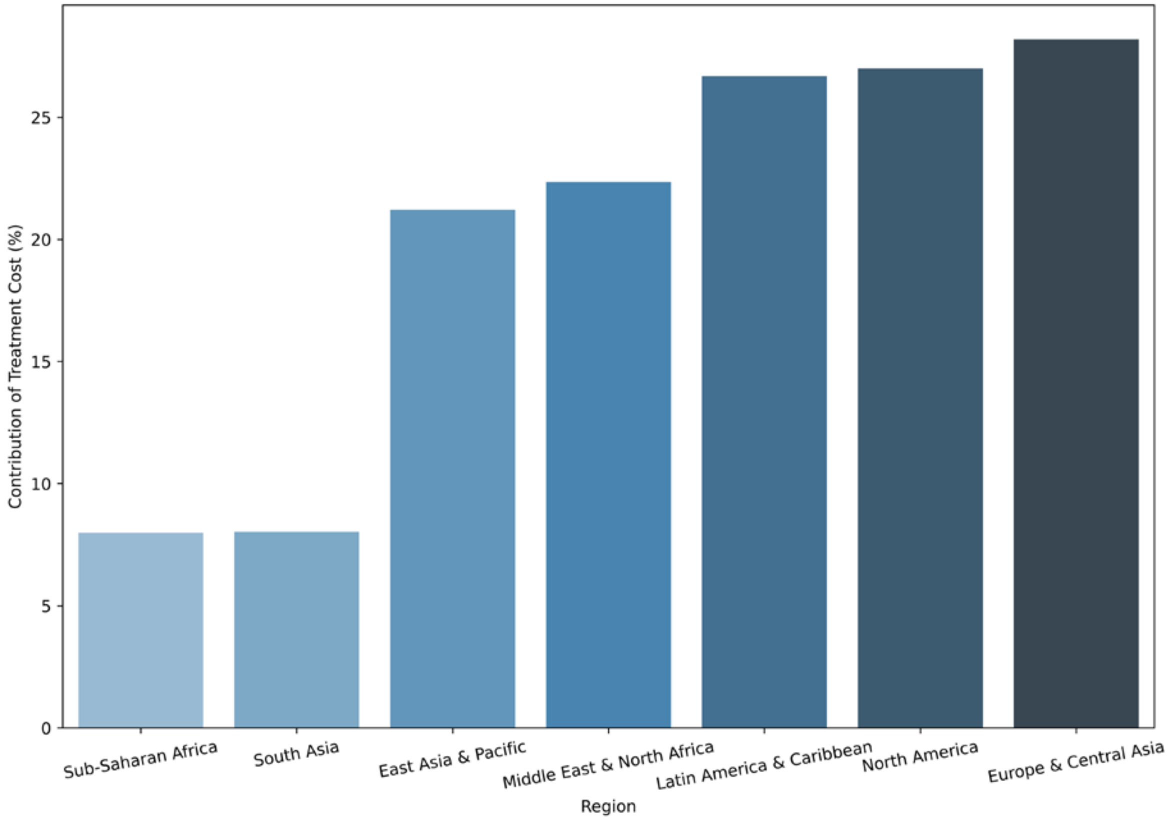

Figure S6. Contribution of treatment costs to the total economic loss due to COPD by World Bank region

H: Strengths and limitations

Strengths

- The calculations are based on recently developed methods and rely on the best available global data, including data from the recently updated Global Burden of Disease Study 2019, the Barro-Lee education database, the World Bank, and the International Labour Organization.
- This study is the first to account for COPD's influence on economic growth through mortality, morbidity, and the effect of treatment expenditures for 204 countries and territories.
- Our framework is the first to consider economic adjustment mechanisms (substitution for labor lost through COPD and savings responses to COPD healthcare costs) explicitly in the analysis for 204 countries and territories. Lack of such consideration has been a key limitation of previous studies.
- This study is the first to incorporate age-specific human capital to account for education-related productivity differences among members of different cohorts who are differentially affected by COPD for 204 countries and territories.
- Our work shows the causal relationship between COPD and GDP. It avoids issues of reverse causality because we did not estimate the relationship but constructed it from our simulated production function.
- We provided a detailed, step-by-step description of our methods and the data sources and specific parameters we used in our analysis in the Appendix.
- We included sensitivity analyses to account for underlying uncertainty by applying varying discount rates (0%, 2%, 3%, 4%, and 5%), by adjusting the mortality and morbidity data based on the upper and lower bounds of the GBD data, and by randomly sampling model parameters (50% to 150% of the initial values).
- We calculated and compared the lifetime health burden with the economic burden to show global inequalities.

Limitations

- To derive country-specific COPD health expenditure we had to extrapolate COPD-related treatment costs for most countries without direct data under the assumption that per case costs are proportional to per capita health expenditure. Specifically, we calculated COPD treatment costs per case for the United States, which are then projected to other countries based on GBD disease prevalence data and health expenditure per capita. This could either underestimate or overestimate country-specific treatment costs for COPD.
- Due to missing data, we had to impute the economic burden of COPD for 60 out of the 204 countries and territories. However, this does not significantly compromise our results, given that the 144 countries for which we had complete data account for 93% of global population and 96% of global GDP.
- We did not include behavioural changes—such as changing labour force participation—among family members who might need to care for patients with COPD. Thus, along this dimension, our findings provide a lower bound for the economic costs of COPD because we did not account for the full costs of patient care.[30]

I: CHEERS guideline

We also included the Consolidated Health Economic Evaluation Reporting Standards 2022 (CHEERS 2022) statement[31] as follows.

Section	*Topic/Checklist item*	*Item No*	*Guidance for reporting*	*Reported on page No/line No*
**Title**	Title	1	Identify the study as an economic evaluation and specify the interventions being compared.	Page 1, Line 1–3
**Abstract**	Abstract	2	Provide a structured summary that highlights context, key methods, results, and alternative analyses.	Page 2, Line 26–48
**Introduction**	Background and objectives	3	Give the context for the study, the study question, and its practical relevance for decision making in policy or practice.	Page 6–7, Line 107–144
**Methods**	Health economic analysis plan	4	Indicate whether a health economic analysis plan was developed and where available.	Page 7–8, Line 148–168; Appendix B
	Study population	5	Describe characteristics of the study population (such as age range, demographics, socioeconomic, or clinical characteristics).	Page 8, Line 171; Appendix C
	Setting and location	6	Provide relevant contextual information that may influence findings.	Page 8, Line 171; Appendix C
	Comparators	7	Describe the interventions or strategies being compared and why chosen.	Page 8, Line 164–168
	Perspective	8	State the perspective(s) adopted by the study and why chosen.	Page 6–7, Line 125–136; Appendix B

	Time horizon	9	State the time horizon for the study and why appropriate.	Page 7, Line 140–142
	Discount rate	10	Report the discount rate(s) and reason chosen.	Page 9, Line 164–168; Appendix C
	Selection of outcomes	11	Describe what outcomes were used as the measure(s) of benefit(s) and harm(s).	Page 8, Line 164–168; Appendix B
	Measurement of outcomes	12	Describe how outcomes used to capture benefit(s) and harm(s) were measured.	Page 8, Line 164–168; Appendix B
	Valuation of outcomes	13	Describe the population and methods used to measure and value outcomes.	Appendix B
	Measurement and valuation of resources and costs	14	Describe how costs were valued.	NA
	Currency, price date, and conversion	15	Report the dates of the estimated resource quantities and unit costs, plus the currency and year of conversion.	Page 9, Line 183–185; Appendix C
	Rationale and description of model	16	If modelling is used, describe in detail and why used. Report if the model is publicly available and where it can be accessed.	Page 6–7, Line 125–136; Appendix B
	Analytics and assumptions	17	Describe any methods for analysing or statistically transforming data, any extrapolation methods, and approaches for validating any model used.	Appendix D
	Characterizing heterogeneity	18	Describe any methods used for estimating how the results of the study vary for subgroups.	Page 9, Line 192–201
	Characterizing distributional effects	19	Describe how impacts are distributed across different individuals or adjustments made to reflect priority populations.	Page 8, Line 171–185; Appendix C
	Characterizing uncertainty	20	Describe methods to characterise any sources of uncertainty in the analysis.	Page 9, Line 192–201

	Approach to engagement with patients and others affected by the study	21	Describe any approaches to engage patients or service recipients, the general public, communities, or stakeholders (such as clinicians or payers) in the design of the study.	NA
**Results**	Study parameters	22	Report all analytic inputs (such as values, ranges, references) including uncertainty or distributional assumptions.	Page 9, Line 192–201; Appendix C; Appendix F
	Summary of main results	23	Report the mean values for the main categories of costs and outcomes of interest and summarise them in the most appropriate overall measure.	Page 10–14, Line 229–234
	Effect of uncertainty	24	Describe how uncertainty about analytic judgments, inputs, or projections affect findings. Report the effect of choice of discount rate and time horizon, if applicable.	Appendix D; Appendix E; Appendix F
	Effect of engagement with patients and others affected by the study	25	Report on any difference patient/service recipient, general public, community, or stakeholder involvement made to the approach or findings of the study.	NA
**Discussion**	Study findings, limitations, generalizability, and current knowledge	26	Report key findings, limitations, ethical or equity considerations not captured, and how these could affect patients, policy, or practice.	Page 18–21, Line 292–376; Appendix G
**Other relevant information**	Source of funding	27	Describe how the study was funded and any role of the funder in the identification, design, conduct, and reporting of the analysis.	Page 9–10, Line 203–211;
	Conflicts of interest	28	Report authors conflicts of interest according to journal or International Committee of Medical Journal Editors requirements.	Page 21, Line 392–393

References

1. Vos T, Lim SS, Abbafati C, et al. Global burden of 369 diseases and injuries in 204 countries and territories, 1990-2019: a systematic analysis for the Global Burden of Disease Study 2019. *The Lancet* 2020; **396**(10258): 1204-22.
2. Bloom DE, Chen S, Kuhn M, McGovern ME, Oxley L, Prettner K. The economic burden of chronic diseases: Estimates and projections for China, Japan, and South Korea. *The Journal of the Economics of Ageing* 2020; **17**(2020): 10016.
3. Chen S, Kuhn M, Prettner K, Bloom DE. The macroeconomic burden of noncommunicable diseases in the United States: Estimates and projections. *PLOS ONE* 2018; **13**(11): e0206702.
4. Bloom DE, Chen S, Kuhn M, Prettner K. The flip side of "live long and prosper": Noncommunicable diseases in the OECD and their macroeconomic impact. In: Bloom DE, editor. Live Long and Prosper? The Economics of Ageing Populations. London, UK: VoxEU.org and Centre for Economic Policy Research (CEPR); 2019. p. 44.
5. Chen S, Kuhn M, Prettner K, Bloom DE. The global macroeconomic burden of road injuries: estimates and projections for 166 countries. *The Lancet Planetary Health* 2019; **3**(9): e390-e8.
6. Chen S, Cao Z, Prettner K, et al. Estimates and Projections of the Global Economic Cost of 29 Cancers in 204 Countries and Territories From 2020 to 2050. *JAMA Oncol* 2023; **9**(4): 465-72.
7. Chen S, Kuhn M, Prettner K, Bloom DE. Noncommunicable Diseases Attributable To Tobacco Use In China: Macroeconomic Burden And Tobacco Control Policies. *Health Affairs* 2019; **38**(11): 1832-9.
8. Chen S, Bloom DE. The macroeconomic burden of noncommunicable diseases associated with air pollution in China. *PLOS ONE* 2019; **14**(4): e0215663.
9. Lucas RE. On the mechanics of economic development. *Journal of monetary economics* 1988; **22**(1): 3-42.
10. Abegunde D, Stanciole A. An estimation of the economic impact of chronic noncommunicable diseases in selected countries. Geneva, Switzerland: World Health Organization, Department of Chronic Diseases and Health Promotion; , 2006.
11. Solow RM. A contribution to the theory of economic growth. *The Quarterly Journal of Economics* 1956; **70**(1): 65–94.
12. Mincer J. Schooling, experience, and earnings. Human Behavior & Social Institutions No. 2. 261 Madison Ave., New York, New York 10016: National Bureau of Economic Research Inc.; 1974.
13. Barro RJ, Lee JW. A new data set of educational attainment in the world, 1950–2010. *Journal of Development Economics* 2013; **104**(September 2013): 184–98.
14. World Bank. World Bank database, GDP, PPP (constant 2017 international $). 2022. https://data.worldbank.org/indicator/NY.GDP.MKTP.PP.KD (accessed April 25 2022).
15. International Monetary Fund. World Economic Outlook Database 2022. 2022. https://www.imf.org/external/pubs/ft/weo/2017/01/weodata/download.aspx (accessed April 25 2022).
16. University of Groningen and University of California. Share of Labour Compensation in GDP at Current National Prices for United States [LABSHPUSA156NRUG], retrieved from FRED, Federal Reserve Bank of St. Louis Davis. 2021. https://fred.stlouisfed.org/series/LABSHPUSA156NRUG (accessed Jan 10 2021).
17. Jones CI. R&D-based models of economic growth. *Journal of political Economy* 1995; **103**(4): 759-84.
18. International Labour Organization. Labour force by sex and age (thousands). http://ilo.org/global/statistics-and-databases/lang--en/index.htm (accessed Sept 1 2020).
19. World Bank. World Bank database, gross savings (% of GDP). 2020. https://data.worldbank.org/indicator/NY.GNS.ICTR.ZS (accessed Sept 1 2020).
20. Dieleman JL, Cao J, Chapin A, et al. US Health Care Spending by Payer and Health Condition, 1996-2016. *JAMA* 2020; **323**(9): 863.
21. Bloom DE, Cafiero E, Jané-Llopis E, et al. The global economic burden of noncommunicable diseases. Geneva, Switzerland: World Economic Forum, 2011.
22. Ding D, Lawson KD, Kolbe-Alexander TL, et al. The economic burden of physical inactivity: a global analysis of major non-communicable diseases. *The Lancet* 2016; **388**(10051): 1311-24.
23. Neumann PJ, Ganiats TG, Russell LB, Sanders GD, Siegel JE. Cost-Effectiveness in Health and Medicine. Oxford University Press; 2016.
24. Haacker M, Hallett TB, Atun R. On discount rates for economic evaluations in global health. *Health Policy and Planning* 2019; **35(1)**: 107–14.

25. Attema AE, Brouwer WBF, Claxton K. Discounting in Economic Evaluations. *PharmacoEconomics* 2018; **36**(7): 745-58.
26. Psacharopoulos G, Patrinos HA. Returns to investment in education: a decennial review of the global literature. *Education Economics* 2018; **26**(5): 445-58.
27. Heckman JJ, Lochner LJ, Todd PE. Earnings functions, rates of return and treatment effects: The Mincer equation and beyond. *Handbook of the Economics of Education* 2006; **1**(2006): 307–458.
28. Grossmann V, Steger T, Trimborn T. Dynamically optimal R&D subsidization. *Journal of Economic Dynamics and Control* 2013; **37**(3): 516-34.
29. World Bank. Life expectancy at birth, total (years). 2022. https://data.worldbank.org/indicator/SP.DYN.LE00.IN (accessed October 13 2022).
30. Cao Z, Chen S. Innovative methods of determining health expenditure efficiency are ur gently needed. *Lancet Glob Health* 2023; **11**(6): e797-e8.
31. Husereau D, Drummond M, Augustovski F, et al. Consolidated Health Economic Evaluation Reporting Standards 2022 (CHEERS 2022) Statement: Updated Reporting Guidance for Health Economic Evaluations. *Value Health* 2022; **25**(1): 3-9.
